# Circumventing the stability problems of graphene nanoribbon zigzag edges

**DOI:** 10.1038/s41557-022-01042-8

**Published:** 2022-09-26

**Authors:** James Lawrence, Alejandro Berdonces-Layunta, Shayan Edalatmanesh, Jesús Castro-Esteban, Tao Wang, Alejandro Jimenez-Martin, Bruno de la Torre, Rodrigo Castrillo-Bodero, Paula Angulo-Portugal, Mohammed S. G. Mohammed, Adam Matěj, Manuel Vilas-Varela, Frederik Schiller, Martina Corso, Pavel Jelinek, Diego Peña, Dimas G. de Oteyza

**Affiliations:** 1 Donostia International Physics Center, San Sebastián, Spain; 2 Centro de Física de Materiales (MPC), CSIC-UPV/EHU, San Sebastián, Spain; 3 Institute of Physics, Czech Academy of Sciences, Prague, Czech Republic; 4 Centro Singular de Investigación en Química Biolóxica e Materiais Moleculares (CiQUS) and Departamento de Química Orgánica, Universidade de Santiago de Compostela, Santiago de Compostela, Spain; 5 Regional Centre of Advanced Technologies and Materials, Czech Advanced Technology and Research Institute (CATRIN), Palacký University Olomouc, Olomouc, Czech Republic; 6 Faculty of Nuclear Sciences and Physical Engineering, Czech Technical University in Prague, Prague, Czech Republic; 7 Ikerbasque, Basque Foundation for Science, Bilbao, Spain; 8 Nanomaterials and Nanotechnology Research Center (CINN), CSIC-UNIOVI-PA, El Entrego, Spain

**Keywords:** Electronic materials, Synthesis and processing, Electronic properties and materials, Scanning probe microscopy

## Abstract

Carbon nanostructures with zigzag edges exhibit unique properties—such as localized electronic states and spins—with exciting potential applications. Such nanostructures however are generally synthesized under vacuum because their zigzag edges are unstable under ambient conditions: a barrier that must be surmounted to achieve their scalable integration into devices for practical purposes. Here we show two chemical protection/deprotection strategies, demonstrated on labile, air-sensitive chiral graphene nanoribbons. Upon hydrogenation, the chiral graphene nanoribbons survive exposure to air, after which they are easily converted back to their original structure by annealing. We also approach the problem from another angle by synthesizing a form of the chiral graphene nanoribbons that is functionalized with ketone side groups. This oxidized form is chemically stable and can be converted to the pristine hydrocarbon form by hydrogenation and annealing. In both cases, the deprotected chiral graphene nanoribbons regain electronic properties similar to those of the pristine nanoribbons. We believe both approaches may be extended to other graphene nanoribbons and carbon-based nanostructures.

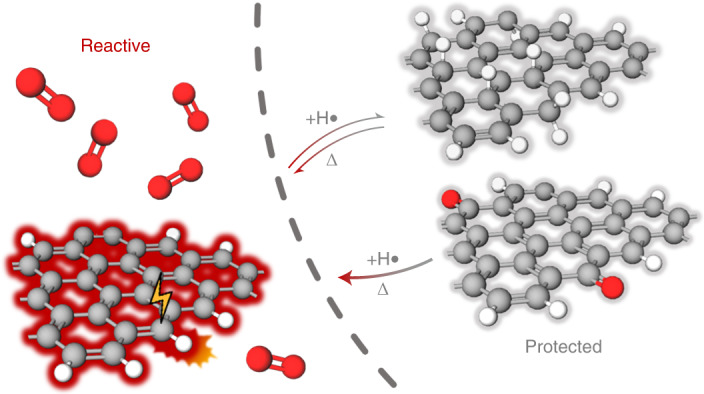

## Abstract (web summary)

On-surface synthesis enables highly reactive structures to be produced under vacuum, but they need to be passivated to be incorporated into practical devices. Here, the facile protection of air-sensitive chiral graphene nanoribbons has been shown, by either hydrogenation or synthesis of an oxidized form. The chemically stable forms can subsequently be deprotected.

## Main

Graphene nanoribbons (GNRs) are very versatile materials. Adjusting their length, width, edge structure or heteroatom dopants can have important effects on their electronic properties, which may vary from being metallic to semiconducting, shifting the band structure up and down in energy or even endowing the nanoribbons with magnetic properties^1–3^. The possibilities further multiply with heterostructures, formed both out of different GNRs^4–6^, as well as in combination with other functional carbon-based materials^7–9^. As a result, GNRs are extremely promising building blocks for a multitude of applications, including quantum technologies. However, some of the most attractive properties of GNRs rely on the presence of zigzag edge segments^10,11^, which, as opposed to armchair edges^12,13^, lack sufficient chemical stability to withstand air exposure even for structures with a dominantly closed-shell character^14^. This severely jeopardizes their potential utilization in actual devices, resulting in a need to conceive alternative strategies for the device implementation process.

Edge groups have been often utilized with GNRs to make them soluble^15,16^ or to tune the ribbon’s electronic properties^17–19^. However, although frequently used in solution chemistry with smaller molecules^20,21^, to date their use for protection purposes on GNRs has remained at a theoretical level^22–24^. In addition, it remains to be tested whether such chemical protection strategies can also be applied to on-surface synthesis^25–27^, which is the approach by which most of the carbon-based nanostructures with peripheral zigzag edges have been synthesized to date^28,29^. Interestingly, such zigzag edges often appear passivated by extra hydrogen atoms^30,31^, causing a rehybridization of the associated carbon atoms into an *sp*^3^ configuration and increasing the structure’s stability. However, it has also been shown that hydrogenation can be used for edge modification of nanoribbons; in particular, it has been used to remove peripheral chlorine^32^ as well as sulfur atoms^33^, with tweaks to the temperature and hydrogen exposure during different stages of the GNR growth also altering their length and termination^33^.

**Fig. 1:**
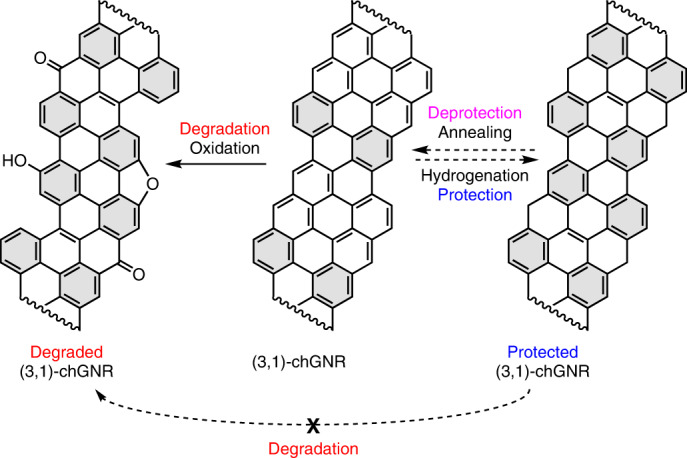
Schematic representation of the degradation of (3,1)-chGNRs exposed to air and the prospective protection strategy by controlled hydrogenation. Hydrogenation of the most reactive carbon atoms at the centre of each zigzag segment^14^ would confer on the ribbons two extra Clar sextets (marked in grey) per unit cell; these are typically associated with larger bandgaps and increased stabilities.

This raises the question of whether an intentional hydrogenation of otherwise air-sensitive GNRs, like chiral nanoribbons displaying a regular alternation of three zigzag and one armchair unit along their edges ((3,1)-chGNRs, where chGNR is chiral graphene nanoribbon)^14^, may act as a protective functionalization that could eventually be controllably removed, for example, by annealing treatments (Fig. 1). In this work, we indeed demonstrate the usage of atomic hydrogen as a means of protecting (3,1)-chGNRs from the oxidizing effects of the atmosphere. A closely related approach further allows converting an air-stable, chemically modified form of the chGNRs with protective ketone side groups back to pristine nanoribbons. Using bond-resolving scanning tunnelling microscopy (BR-STM) and non-contact atomic force microscopy (nc-AFM), along with scanning tunnelling spectroscopy, X-ray photoemission spectroscopy (XPS) and density functional theory (DFT) calculations, we show that both chemically protected forms of the GNRs (hydrogenated and ketone functionalized) survive exposure to air. The GNRs that follow the deprotection processes are mostly pristine, with their electronic properties remaining intact.

## Results

### Hydrogenation for protection and annealing for deprotection

**Fig. 2:**
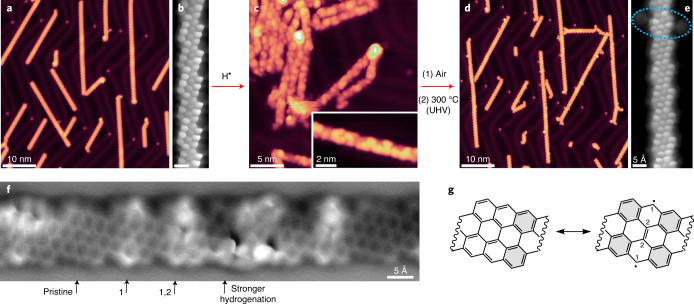
Scanning probe microscopy analysis of GNRs along the various steps in the cycle of synthesis, hydrogenation (protection), air exposure and annealing (deprotection). **a**, Overview STM image of the sample of pristine (3,1)-chGNRs on Au(111) prior to hydrogenation. Scanning parameters: *I* = 1 nA, *U* = −0.5 V. **b**, BR-STM image of a pristine GNR (CO tip, constant height, *U* = 5 mV; scale bar, 500 pm). **c**, STM image of a cluster of hydrogenated ribbons after exposure to atomic hydrogen (H·). The inset shows a zoomed-in view of a hydrogenated nanoribbon, showing strong changes in contrast in sections with different levels of hydrogenation. For both images, *I* = 23 pA and *U* = −2.0 V. **d**, Overview STM image of the hydrogenated nanoribbons after 24 minutes of air exposure followed by annealing to 300 °C in ultra-high vacuum (UHV). *I* = 20 pA, *U* = −0.5 V. The image shows the recovery of quasi-unaffected pristine GNRs and thus the successful completion of a protection/deprotection cycle. **e**, BR-STM image of a mostly pristine nanoribbon after the same treatment, with one defective/oxidized section with metal-coordinated ketone groups circled (CO tip, constant height, *U* = 5 mV). **f**, A nc-AFM image of a weakly hydrogenated chGNR. The arrows mark representative unit cells that remain pristine, hydrogenate at position ‘1’, hydrogenate at positions ‘1’ and ‘2’, and show stronger hydrogenation. **g**, Closed-shell (left) and open-shell (right) resonant forms of a chGNR unit cell, displaying for the latter the positions most prone (‘1’) and second most prone (‘2’) to becoming hydrogenated, in direct relation to the strongest radical character for ‘1’ and the atoms whose rehybridization does not affect Clar sextets in the case of ‘2’. Source data

The synthesis of chGNRs on a Au(111) surface was performed according to previous protocols^34,35^, via annealing 2,2′-dibromo-9,9′-bianthracene (chiral DBBA) precursors up to 350 °C. Following this, the resulting nanoribbons were examined via BR-STM to ensure that they were pristine (denoted henceforth as p-chGNRs). Representative images of the pristine ribbons are shown in Fig. 2a,b. To protect the edges, the GNRs were exposed, with the sample held at room temperature, to a flux of atomic hydrogen originated from a molecular H_2_ flow through a heated tungsten tube (Methods for details). Following this treatment, the GNRs were examined again at 4 K by STM measurements. A representative STM image of the sample after this GNR protection is shown in Fig. 2c. Notably, substantial changes in the apparent height contrast within the ribbons are observed, as can be seen best in the inset of Fig. 2c. Supplementary Fig. 1 shows a comparison between the apparent height profile of pristine and hydrogenated ribbons, with the ribbons approximately doubling their apparent height after hydrogenation. This implies that a notable number of the carbons are converted from *sp*^2^ to *sp*^3^ upon hydrogen exposure, adopting a non-planar conformation to accommodate the change in favoured bond angles. A comparison to previous reports in the literature, in which some of the edge carbons of chGNRs were shown to be hydrogenated but did not show substantial increases in their height profile^14,30^, leads to the conclusion that several internal double bonds of the GNR backbone have also been hydrogenated, leading to sections of ‘graphane’ nanoribbon that notably differ from the idealized picture in Fig. 1 and display extra hydrogen atoms pointing out of the plane of the molecular backbone. Furthermore, this conversion is not uniform, as can be seen from the topographic images in Fig. 2c and the aforementioned height profiles.

Because such strongly hydrogenated GNRs as those shown in Fig. 2c cannot be adequately imaged with bond-resolving power, to get an atomistic picture of the hydrogenation, we have also characterized ribbons that are only lightly hydrogenated (Methods for details). A representative nc-AFM image is shown in Fig. 2f, which shows the presence of unit cells that remain unaffected (labelled as pristine), along with others that are subject to different degrees of hydrogenation. Pristine chGNRs have been shown to display a predominantly closed-shell character^36,37^. However, a minor contribution of an open-shell resonant form as pictured in Fig. 2g is considered responsible for the notable reactivity of these ribbons^14^. For bisanthene molecules (that is, the chGNR unit cell) their open-shell resonant form contributes 12% (ref. ^38^), and it is expected to be in the same order of magnitude for the ribbons considered here. It is intuitive to expect the radical positions (labelled as ‘1’) to be most reactive. DFT calculations indeed confirm that those are the energetically most favourable sites for initial hydrogenation (Supplementary Fig. 2 and Supplementary Fig. 3). Thereafter, the inner unit cell atoms (labelled as ‘2’) are the only other carbon atoms whose *sp*^3^ rehybridization does not destroy any of the stabilizing Clar sextets that are marked in grey in Fig. 2g (ref. ^39^). In line with this reasoning, lightly affected unit cells display a clear prevalence for initial hydrogenation at the ‘radical sites’ (‘1’), subsequent hydrogenation at the inner unit cell atoms (‘2’) and higher hydrogenation levels at the remaining C atoms. Examples for each of these three cases can be observed in the unit cells labelled as ‘1’, ‘1,2’ and ‘stronger hydrogenation’ in Fig. 2f.

The p-chGNRs, which typically appear regularly dispersed over the surface^35^, exhibit a pronounced tendency to aggregate together after the hydrogen exposure. The acquired non-planarity lowers their interaction with the underlying surface^40^ and at the same time promotes the van der Waals interactions between the GNRs, altogether leading to a favoured island formation. Also the interface dipole between the GNRs and the surface may be affected. For low bandgap GNRs like the pristine ones shown here, Fermi level pinning has set in^41^. This is associated with a partial charge transfer from the ribbon to the Au, which sets up a notable interface dipole and may cause repulsion between adjacent GNRs. As the ribbons hydrogenate, their bandgap widens and the charge transfer vanishes, and thereby also the repulsive dipole–dipole interactions vanish.

To test the air stability of the hydrogenated nanoribbons, the sample was next taken out of its ultra-high vacuum environment and exposed to ambient conditions for 24 minutes (Methods). Following this, it was transferred back to ultra-high vacuum (pressure in the low 10^−10^ mbar range) and annealed to 300 °C for 20 minutes to both dehydrogenate (that is, ‘deprotect’) the GNRs and remove coadsorbed contaminants from the air. STM images after this treatment are shown in Fig. 2d,e. The vast majority, namely ~90%, of the chGNR units cells are found to be pristine (561 out of 623 analysed unit cells) and also clearly retain their electronic properties^36^, as shown in Supplementary Fig. 4 with undisturbed conductance maps at the valence and conduction band onsets. A few defects are observed—most commonly, the central ring of the zigzag edge appears larger in BR-STM imaging, with a sharp point at the edge that is oriented towards a feature that presumably corresponds to a gold adatom (Supplementary Fig. 5). This is similar to the most commonly observed defect during oxidation experiments of p-chGNRs, which has been assigned to a ketone that is coordinated to a gold adatom^14^. An example of such a defect is circled in Fig. 2e, with a pair of these ketones on the same GNR unit cell. As the number of defects is very low, this result clearly demonstrates the effectiveness of the hydrogenation strategy in protecting the GNRs from the oxidizing effect of the atmosphere; previous experiments observed substantial levels of oxidation even at low pressures (10^−5^ mbar) of pure molecular oxygen when exposing the p-chGNRs^14^.

**Extended Data Fig. 1:**
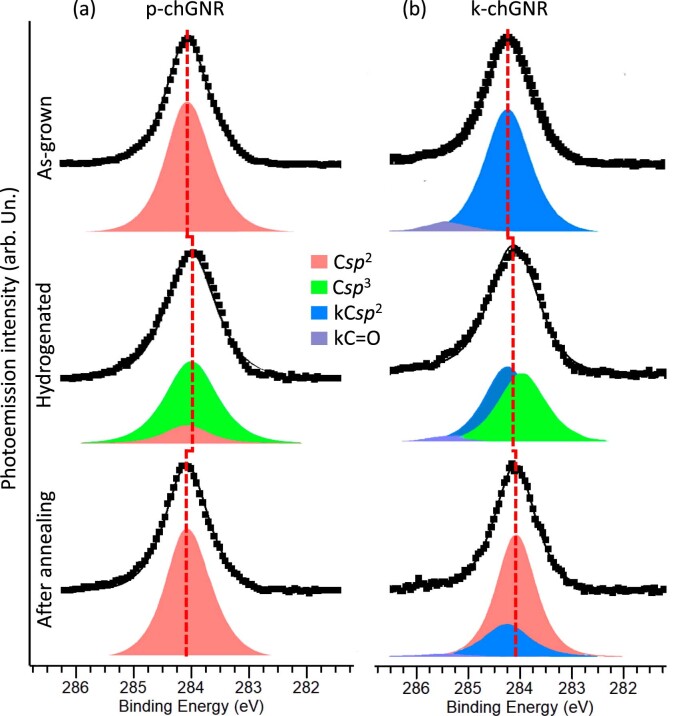
XPS analysis monitoring the hydrogenation/dehydrogenation process of pristine and ketone-functionalized ribbons. (a) C1s XPS spectra (symbols) of p-chGNRs “as grown” (top), after hydrogenation (middle) and after subsequent annealing (bottom), along with the fit (solid curve) and its associated components (coloured peaks). The data reveal the successful hydrogenation and its reversibility. (b) C1s XPS spectra (symbols) of k-chGNRs “as grown” (top), after hydrogenation (middle) and after subsequent annealing (bottom), along with the fit (solid curve) and its associated components (coloured peaks). The data reveal the gradual deoxidation upon hydrogenation and the subsequent annealing. Dashed lines act as a guide to the eye to compare the maxima on each of the spectra. Details about the fitting procedure are given in the methods section. Source data

The whole hydrogenation (protection) and dehydrogenation (deprotection) process was additionally investigated by XPS, as summarized in Extended Data Fig. 1. Although the resolution of our laboratory X-ray source does not allow us to resolve individual components of the various carbon species in the GNRs in their pristine and hydrogenated forms, there is a clear broadening towards the low binding energy side for the latter. A lower binding energy is indeed expected from the hydrogenated *sp*^3^ carbon atoms^42^, in line with our observations. Most importantly, the initial spectral shape is recovered after the annealing treatment, confirming the reversibility and effectiveness of the protection/deprotection process (Extended Data Fig. 1).

### Pre-oxidized nanoribbons

**Fig. 3:**
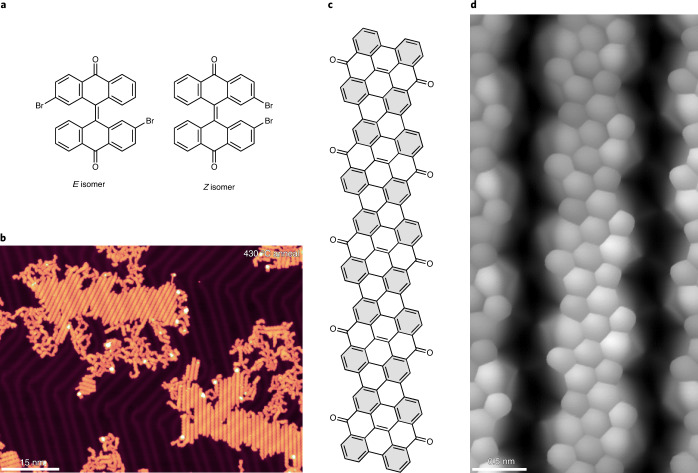
Reactant and product structure of pre-oxidized (protected) chGNRs. **a**, Structures of the two k-DBBA precursor isomers. **b**, Overview STM image of self-assembled islands of ketone chiral nanoribbons after annealing the precursors in steps up to 430 °C. Scanning parameters are *I* = 30 pA, *U* = −0.5 V. **c**, Chemical structure of the k-chGNRs, with Clar sextets marked by a grey background. The increase from two to four Clar sextets when comparing pristine GNRs and these pre-oxidized GNRs supports the greater stability of the latter. **d**, BR-STM image of a ketone chiral nanoribbon after air exposure and post-annealing to 200 °C in ultra-high vacuum conditions. The sharp features at the edge of each central ring correspond to the ketone groups. No changes are observed before and after the air exposure (CO tip, constant height, *U* = 8 mV), showing the air-stable character of the nanoribbons. Source data

**Extended Data Fig. 2:**
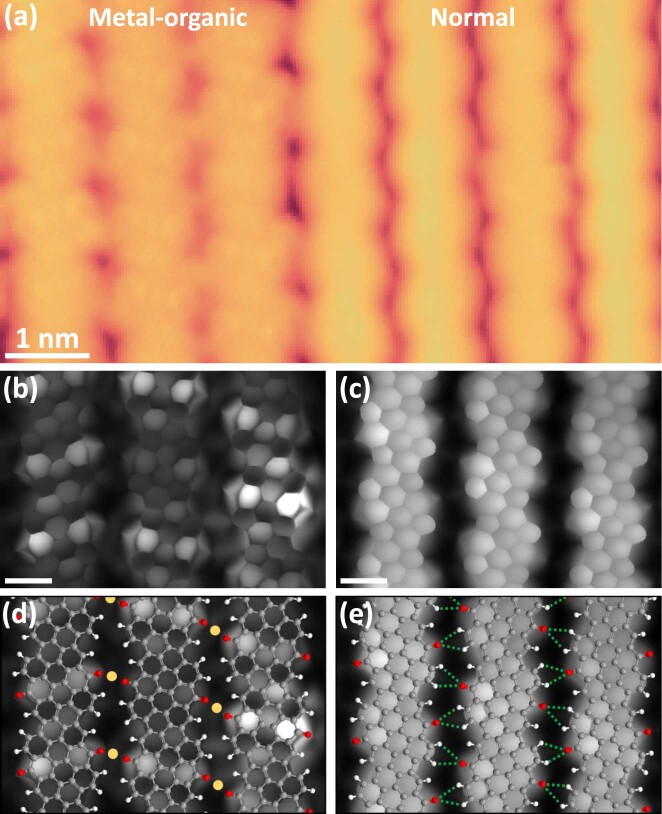
Comparison of ‘typical’ and ‘metal-copordinated’ k-chGNRs. Comparison between the structures of the two types of ketone-functionalized GNRs that were observed. (a) Typical STM image of a self-assembled island that contains both types of ketone-GNR. I = 50 pA, V_bias_ = −0.5 V. (b) BR-STM image of a metal-coordinated and (c) typical section of a ketone-NR island. Both constant height, CO tip, V_bias_ = 4 mV and 5 mV, respectively. Scale bars are both 500 pm. (d) and (e) Models of the metal-coordinated and typical islands, with suggested positions for gold adatoms in the metal-coordinated model. Source data

To further understand the tendency of chGNRs to oxidize at their zigzag edges, we have utilized a different precursor molecule: 2,2′-dibromo-10*H*,10′*H*-(9,9′-bianthracenylidene)-10,10′-dione ((*E*/*Z*)-k-DBBA; Fig. 3a; Supplementary Information for synthetic details). This molecule is already ‘pre-oxidized’ with ketone groups bound at its central zigzag edge positions, which were previously found to be the most reactive sites^14^. The self-assembly of a mixture of *E* and *Z* isomers of k-DBBA is presented in Supplementary Fig. 6. Annealing this precursor mixture on Au(111) to relatively high temperatures (430 °C) results in the formation of nanoribbons, henceforth referred to as ketone-chGNRs (k-chGNRs). As shown in Fig. 3b, these nanoribbons readily pack together into islands. The chemical structure of the k-chGNRs is shown in Fig. 3c. Two different forms of these nanoribbons are observed: (1) ‘typical’ k-chGNRs, which are found in islands held together by hydrogen bonds; and (2) ‘metal-coordinated’ k-chGNRs, found in islands stabilized by apparent metal–organic coordination bonds. In the case of the latter, the ketone groups are observed pointing towards each other, implying the presence of a shared metal atom, whereas in the former, the ketones are oriented towards the hydrogen atoms of an adjacent ribbon. Both forms of nanoribbons are shown in model structures in Extended Data Fig. 2 for comparison; the tentative hydrogen bonds with an average length of 2.3 ± 0.2 Å are also indicated. Although both types of nanoribbons are always present, their ratio varies depending on the exact annealing procedure used during their formation. A fast annealing straight to a high temperature typically yields mostly metal-coordinated GNRs, whereas a slow or stepwise annealing (370 °C, 400 °C and 430 °C) yields more ‘typical’ k-chGNRs. In all experiments, there was a notable issue with anthrone-related molecules (described in further detail in Supplementary Fig. 7). These molecules are often found at the termination of the k-chGNRs and may be the cause of their relatively short average length when compared to p-chGNRs that are formed from chiral DBBA. We hypothesize that the *Z* isomer, which does not form GNRs, may be the cause of this contamination. It may terminate the polymers and fragment when heated on the surface. Future studies with stereoisomerically pure precursors^35^ may clarify this issue and improve the length and quality of the nanoribbons.

**Extended Data Fig. 3:**
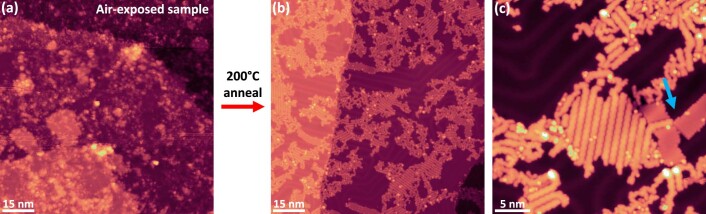
STM analysis of the air exposed k-chGNR sample before and after annealing. (a) Overview STM image of a ketone-GNR sample that was exposed to air for 24 minutes, without any further treatment. I = 50 pA; V_bias_ = 1.5 V. (b) and (c): STM images of the same sample after annealing to 200 °C for 1 hour. (b) I = 20 pA; V_bias_ = −0.8 V. (c) I = 30 pA; V_bias_ = −0.8 V. Although most have desorbed, a notable number of contaminants are still present on the surface after the annealing treatment. Islands of alkyl chain contamination from the ambient conditions are indicated in (c) with a blue arrow. Source data

An example of a BR-STM image of the ‘typical’ type of k-chGNRs is shown in Fig. 3d. Each unit cell exhibits two sharp features located at the centre of its zigzag edge, corresponding to the positions of the ketones. These features match with previously observed oxidation defects in p-chGNRs^14^, as well as those described above after exposing the hydrogenated GNRs to air. Crucially, these pre-oxidized chGNRs are no longer susceptible to oxidation under ambient conditions; after exposing a sample of k-chGNRs to air (24 minutes) and post-annealing (200 °C) to remove coadsorbed contaminants (Extended Data Fig. 3), the vast majority of the k-chGNR units are unaffected, with only a small minority (10%) displaying any extra defects that could be ascribed to the air exposure. Detailed BR-STM images of some of these defects are shown in Supplementary Fig. 8. A clear demonstration of the general air stability of the k-chGNRs is the image shown in Fig. 3d, which was actually recorded after air exposure followed by annealing and displays no changes from typical BR-STM images recorded prior to the exposure.

**Fig. 4:**
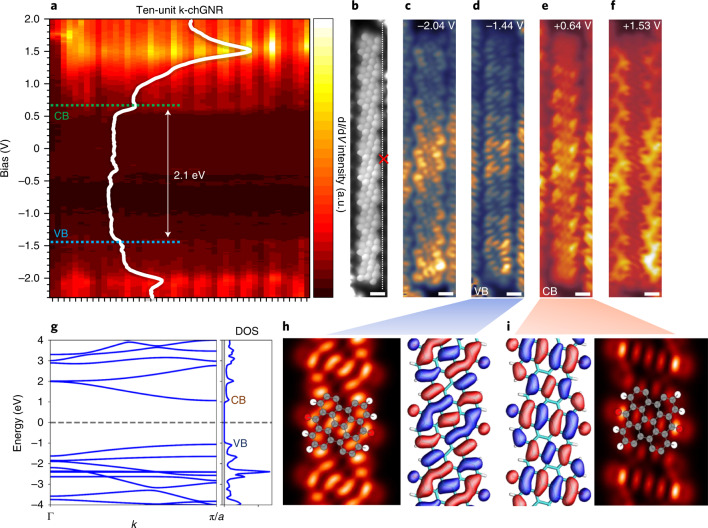
Electronic properties of pre-oxidized protected GNRs. **a**, Stacked d*I*/d*V* point spectra along the edge of a ten-unit ketone nanoribbon. The onsets of the VB and CB are indicated with dashed lines, with a measured bandgap of 2.1 eV. One of the spectra is overlaid with a white line. **b**, BR-STM image of the same ten-unit nanoribbon; the dashed line marks the positions along which the spectra were acquired that make up **a**, and the red cross marks the position for the overlaid spectrum (constant height, CO tip, *U* = 5 mV). **c**–**f**, Constant height d*I*/d*V* images of the four strongest resonances observed in the d*I*/d*V* spectra at –2.04 V (**c**), –1.44 V (**d**), +0.64 V (**e**) and +1.53 V (**f**; CO tip; scale bars, 500 pm). **g**, Band structure calculations for the ketone-functionalized ribbons (aligned with respect to the mid-gap energy, with k denoting the electron momentum and a the unit cell size), along with the integrated density of states (DOS). **h**, Wavefunction (right) and simulated STM image (left) of the orbital at the VB onset. The simulated images consider tips with 95% *p*_*x*_*p*_*y*_-wave and 5% *s*-wave character, and the unit cell structure used for the calculations is superimposed on the images, with grey atoms corresponding to C, red, to O and white, to H. **i**, Similar wavefunction (left) and simulated STM image (right) at the CB onset. Source data

The addition of a ketone group causes notable changes in the conjugation of the chGNRs. As a consequence, the electronic properties are strongly affected. Each unit cell can be represented with four Clar sextets (Fig. 3c), as opposed to the two Clar sextets that may be drawn for the (closed-shell) representations of the pristine (3,1)-chGNRs (Fig. 1). Such an increase is typically associated with an enlarged bandgap and a concomitantly augmented stability. Figure 4a displays stacked d*I*/d*V* spectra (*I*, current; *V*, voltage) recorded at 45 points along the edge of a ‘typical’ ten-unit k-chGNR as shown in Fig. 4b. Overlaid is an example of one of the point spectra, recorded at the position marked with a red cross in Fig. 4b. The onsets of both the valence and conduction bands (VB and CB, respectively) are indicated with dashed lines. Besides conferring to the ribbons an n-type semiconducting character, the ketone functionalization endows the ribbons with a much larger bandgap energy (*E*_g_ ≈ 2.1 eV) than that of the pristine chGNRs (*E*_g_ ≈ 0.67 eV)^36^. As expected, shorter k-chGNRs show a wider bandgap (for example, 2.5 eV for a dimer), but the value very quickly saturates around 2.05–2.10 eV after the chain reaches four or five units in length (Supplementary Fig. 9). This notably wider bandgap supports the assertion of the k-chGNRs’ increased stability with respect to the p-chGNRs. A BR-STM image of the ten-unit ketone nanoribbon, along with four constant-height d*I*/d*V* maps (taken with a CO-functionalized tip), are presented in Fig. 4b–f. These are recorded at the VB and CB onsets, and at two conductance maxima that are found deeper inside the occupied and unoccupied states.

To improve our understanding of the ribbon’s electronic properties, we performed complementary DFT calculations. Figure 4g displays the calculated band structure, along with the integrated density of states. In qualitative agreement with the experiments, the initial density of states maxima at the VB and CB onsets are followed by stronger maxima, out of which the occupied states are closer to the VB onset than the empty states to the CB onset. These two density of states maxima correspond to van Hove singularities at the Brillouin zone centre Γ that contain contributions from the VB and CB in combination with the following bands (as opposed to the VB and CB onsets at the zone boundary). Focusing on the frontier states, the calculated wavefunctions and the associated STM image simulations show an excellent agreement with the measurements and confirm our assignment (Fig. 4h,i).

**Fig. 5:**
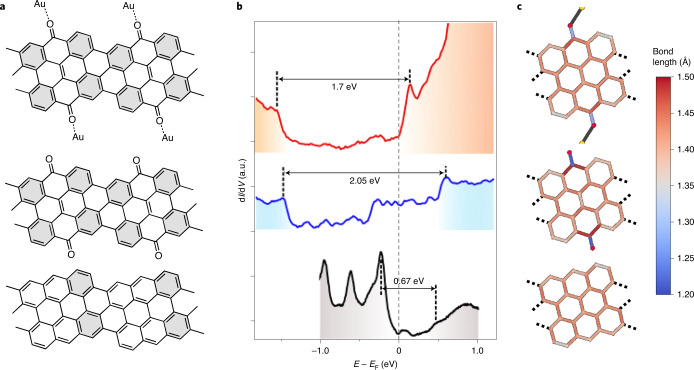
Comparison of the chemical and electronic structures of the various GNRs studied. **a**–**c**, Chemical structure (**a**), representative d*I*/d*V* spectra (**b**) and calculated bond lengths (**c**) of p-chGNRs (bottom), ‘typical’ k-chGNRs (middle) and ‘metal-coordinated’ k-chGNRs (top). The change in the conjugation pattern from pristine to k-chGNRs causes more inhomogeneous bond lengths and increased number of Clar sextets per unit cell at well-defined positions, which augments the structure’s bandgap. The metal coordination weakens the bond length inhomogeneity and thus lowers the bandgap to an intermediate value between that of the ‘typical’ k-chGNRs and that of the p-chGNRs (although closer to the former). In panel (**b**) the energy of the conduction and valence band onsets with respect to the Fermi energy (*E*_F_) are marked with dashed lines and the band gap is depicted with the horizontal arrows. Source data

Although no difference has been observed between the ‘typical’ and the ‘metal-coordinated’ k-chGNRs with regard to their air stability, the electronic properties show some notable changes. Figure 5 compares conductance spectra of typical and metal-coordinated k-chGNRs, along with p-chGNRs for reference. Metal-coordinated k-chGNRs have a reduced bandgap as compared to the typical counterparts, dropping to around 1.7 eV (Fig. 5). The coordination to Au lowers the energy of both VB and CB onsets, with the CB found notably lower (change in energy, Δ*E* ≈ 0.5 eV) than that of the typical k-chGNRs. As a result, the CB gets so close to the Fermi level that its contribution becomes evident in the BR-STM images of metal-coordinated k-chGNRs (Extended Data Fig. 2). This causes a much stronger contrast variation within the ribbon (due to the orbital’s spatial distribution) that is also notably length dependent. Similarly to typical k-chGNRs, shorter ribbons have wider bandgaps and thus a lower contribution of the CB to the BR-STM images. The overall downshift of the bands (that is, of the mid-gap energy) is presumably related to an increased ketone-related electric dipole upon Au coordination, whereas a tentative explanation for the bandgap reduction may lie in the change of the conjugation of the system upon the formation of the metal-coordinated bond. The latter reduces the C=O double-bond character and thereby pushes the system slightly towards the conjugation pattern and the smaller bandgap of the p-chGNRs. DFT calculations on periodic Au-coordinated arrays of k-chGNRs confirm this hypothesis. Figure 5c compares the calculated bond lengths of free-standing chGNRs in the absence of the ketone groups, with ketones and with Au-coordinated ketones, showing the unambiguous tendency of the Au coordination to elongate the C=O bond, reducing its double-bond character and concomitantly modifying the bond lengths and conjugation of the rest of the carbon backbone (the effect on the band structure is displayed in Supplementary Fig. 10). At this point it should be noted that the calculated bond lengths remain virtually unchanged when including the substrate into the calculations (Supplementary Figs. 11 and 12), supporting the same conclusions as those reached from free-standing calculations for these systems. Altogether, the addition of coordinating Au adatoms, whether in the presence or absence of the substrate, nicely reproduces our experimental findings and provides a qualitative understanding of the ‘analyte sensing’ ability of the ketone groups.

### Hydrogenation and annealing for deprotection

**Fig. 6:**
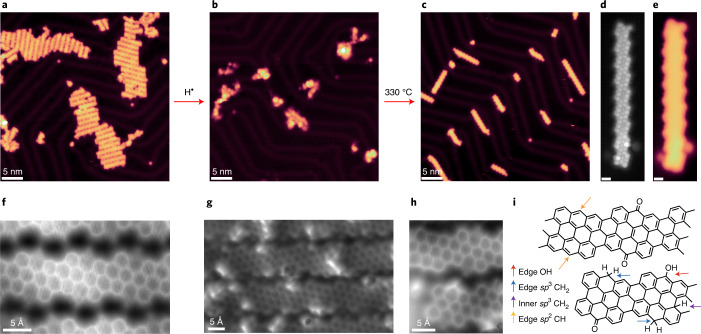
STM analysis of GNRs along the various steps in the synthesis process of its pre-oxidized (protected) form, hydrogenation and annealing (deprotection). **a**, Overview STM image (*I* = 100 pA, *U* = −0.1 V) of mostly metal-coordinated ketone GNRs after their synthesis on Au(111). **b**, Overview STM image (*I* = 50 pA, *U* = −1.0 V) of the same GNR sample after exposure to atomic hydrogen. **c**, STM image (*I* = 50 pA, *U* = −0.5 V) of the same sample after annealing to 330 °C. The GNRs mostly consist of pristine sections, with some remaining ketone defects, proving a successful deprotection of the air-stable k-chGNRs. **d**,**e**, BR-STM (constant height, CO tip, *U* = 5 mV; **d**) and STM (*I* = 100 pA, *U* = −0.5 V; **e**) of one of the GNRs from the post-annealed sample (scale bars, 500 pm). Two neighbouring metal-coordinated ketone defects are observed on the two sections at the lower end of the ribbon. **f**, nc-AFM image of as-grown k-chGNRs. **g**, nc-AFM image of lightly hydrogenated k-chGNRs, showing very inhomogeneous hydrogenation motifs. **h**, Detail of lightly hydrogenated k-chGNRs. **i**, The proposed chemical structure of the detail in panel **h**, which includes various types of products along with deoxidized units. Source data

As previously mentioned, the k-chGNRs are found to be particularly stable (in contrast to the p-chGNRs^14^) due to the presence of carbonyl (C=O) groups that allow the formation of more Clar sextets without the concomitant creation of unpaired radicals. However, disrupting this system with atomic hydrogen may result in the formation of hydroxy derivatives that can in turn be involved in dehydration to afford the p-chGNRs upon annealing. This was tested on samples with both dominant typical and dominant metal-coordinated k-chGNRs (Fig. 6), showing no noticeable difference.

Hydrogenation of the k-chGNRs (Fig. 6b) under similar conditions to the previous experiments (Methods) results in smaller dispersed islands of molecular aggregates, many of which are distorted into small ‘three-dimensional’ objects with little resemblance to nanoribbons. However, annealing this sample to 330 °C clearly demonstrates that the ribbons’ backbones are not destroyed by the hydrogenation process, as elongated, straight structures are once again formed (Fig. 6c). Examination of these structures via BR-STM (Fig. 6d) shows that they are, in fact, now mostly pristine GNRs, with a small number of ketone defects still remaining. Most of them appear coordinated to Au atoms (for example, the two units at the bottom of the GNR in Fig. 6d,e) and in closed-shell defect pair configurations, that is, either on the same or on neighbouring unit cells^43^. These defects have the same appearance as those observed when exposing the hydrogenated pristine GNRs to air^14^. The high level of conversion (~78%, 602 out of 772 unit cells analysed) may be further improved with tweaks to the hydrogenation process or repeated hydrogenation/annealing cycles. However, this result is noteworthy in and of itself. Note that the coverage reduction that may be apparent in Fig. 6 is spurious and reflects only the disparate tendency of k-chGNRs to assemble into compact islands while p-chGNRs repel each other. Larger-area images (Supplementary Fig. 13) show how the low density of compact islands is comparable in coverage to that of the disperse p-chGNRs.

To shed more light onto the whole hydrogenation and annealing process, we performed additional nc-AFM and XPS experiments. The former cannot be performed with intramolecular bond resolution on heavily hydrogenated structures that become exceedingly non-planar. However, the measurement of only lightly hydrogenated ribbons provides insight into the first chemical changes upon hydrogen exposure. A reference nc-AFM image of ‘as-grown’ k-chGNRs is displayed in Fig. 6f, showing the fading contrast of all the bonds involving the ketone-functionalized carbon atom. A representative image of a hydrogenated sample is shown in Fig. 6g. Less reproducible products than those found for the p-chGNR counterparts point towards more complex or competing reaction processes. Nonetheless, several conclusions can be readily drawn. As for p-chGNRs, it is also true for k-chGNRs that the central carbon atom of the zigzag segments (which is the ketone-functionalized atom) is among the most affected. Indeed, as more clearly shown in the smaller scale image in Fig. 6h, the contrast on these atoms displays multiple variations along with that of the starting nanoribbon. Our tentatively proposed assignment, based on the comparison with previously characterized defects^14^, is shown in Fig. 6i and includes hydroxyl-functionalized carbon atoms, as well as deoxidized *sp*^2^ and *sp*^3^ atoms. Complementary DFT calculations show the oxygen atom as the energetically most favourable initial hydrogenation site (Supplementary Fig. 14). Once the hydroxyl group has been formed, the conjugation pattern becomes closer to that of p-chGNRs, and the following hydrogenation events can cause either dehydration (affording p-chGNRs) or the hydrogenation of other carbon atoms. These additional options also justify the larger diversity in hydrogenation products observed experimentally when compared to the initial hydrogenation stages of p-chGNRs.

XPS measurements confirm that a partial deoxidation starts upon hydrogen exposure even before the annealing treatment (Extended Data Fig. 1). Following the fitting procedure described in more detail in the Methods, we infer that right after hydrogenation ~40% of the oxygen atoms in the k-chGNRs are removed from the ribbons. The ratio augments to ~70% after the annealing treatment while maintaining the total amount of carbon signal nearly unaffected and therefore discarding any substantial desorption. Both of these findings are in qualitative agreement with the scanning probe microscopy analysis.

While the GNRs resulting from this process are relatively short in length, this is not related to the hydrogenation process itself, but to the short average length of the k-chGNRs that they began as. Improvements in the synthesis of the k-chGNRs, which may be presumably achieved with a reduced amount of *Z* isomer as well as with optimized growth parameters, should also result in longer, higher quality pristine GNRs after the hydrogenation and annealing processes.

These results demonstrate that chemically protected GNRs can be easily converted back to pristine GNRs. This method could apply to other protecting groups and functionalities that have different properties, as long as the central carbon at the zigzag edge is still present. Reducing the level of hydrogenation may also allow for statistical mixtures of functionalized and pristine GNRs that have different properties.

## Discussion

Hydrogenation cannot necessarily be used as a ‘cure’ for uncontrollably oxidized GNRs, since some of the common defects after exposing chGNRs to air involve modifications of the carbon backbone structure and even the loss of carbon atoms that cannot be recovered through hydrogenation^14^. However, we have shown that this method allows the application of protection/deprotection strategies, as commonly utilized in conventional organic chemistry, to on-surface synthesis. That is, hydrogenation can be used both to protect chemically labile GNRs, as well as to deprotect pre-oxidized nanoribbons that possess a substantially larger bandgap and built-in resistance to oxidizing atmospheres, in either case allowing for a reconversion into (mostly) p-chGNRs. Most importantly, we believe that this approach may be extrapolated to different GNRs and carbon-based nanostructures, as well as to different functional groups that are involved in pre-protected forms, altogether bringing the exploitation of the unique characteristics of zigzag edges in carbon materials a step closer to scalable applications.

## Methods

### Synthesis of k-DBBA

#### General methods for solution synthesis

All reactions were carried out under argon using oven-dried glassware. Thin-layer chromatography (TLC) was performed on Merck silica gel 60 F_254_; chromatograms were visualized with UV light (254 and 360 nm). Flash column chromatography was performed on Merck silica gel 60 (ASTM 230–400 mesh). The ^1^H NMR were recorded at 300 MHz (Varian Mercury 300). Low-resolution electron impact mass spectra were determined at 70 eV on an HP-5988A instrument. High-resolution mass spectra were obtained on a Micromass Autospec spectrometer. Commercial reagents were purchased from ABCR, Aldrich Chemical, and were used without further purification. Tetrahydrofuran (THF) and Et_2_O were purified by a MBraun SPS-800 Solvent Purification System. Anthrone **6** was prepared following a published procedure shown in Supplementary Fig. 15 (ref. ^44^).

#### Synthesis of (*E*/*Z*)-k-DBBA

Over a solution of anthrone **6** (500 mg, 1.84 mmol) in CH_2_Cl_2_ (20 ml), 1,8-Diazabicyclo[5.4.0]undec-7-ene (DBU) (0.97 ml, 6.50 mmol) was added and the mixture was stirred for 10 min. Then, iodine (514 mg, 2.02 mmol) was added and the resulting solution was stirred in the absence of light for 24 h. Then, aqueous HCl (10%, 10 ml) and a saturated aqueous solution of Na_2_S_2_O_3_ (20 ml) were added, the organic layer was separated and the aqueous phase was extracted with CH_2_Cl_2_ (2 × 20 ml). The combined organic extracts were dried over Na_2_SO_4_, filtered and evaporated under reduced pressure. The residue was purified by column chromatography (SiO_2_; CH_2_Cl_2_), affording a mixture of k-DBBA isomers (1:1, 302 mg, 61%) as an orange solid (Supplementary Fig. 16 and 17).

The isomer 1 conditions were as follows: ^1^H NMR (300 MHz, CDCl_3_) chemical shift, *δ*: 8.14 ppm (*d*, coupling constant *J* = 8.0 Hz, ^1^H), 8.00 ppm (*d*, *J* = 8.6 Hz, ^1^H), 7.62 ppm (*dd*, *J* = 8.3, 1.9 Hz, ^1^H), 7.55–7.43 ppm (*m*, ^1^H), 7.31–7.25 ppm (*m*, ^1^H), 7.20 ppm (*d*, *J* = 1.9 Hz, ^1^H) and 7.03 ppm (*t*, *J* = 7.1 Hz, ^1^H).

The isomer 2 conditions were as follows: ^1^H NMR (300 MHz, CDCl_3_) *δ*: 8.10 ppm (*d*, *J* = 7.8 Hz, ^1^H), 7.97 ppm (*d*, *J* = 9.3 Hz, ^1^H), 7.55 ppm (*dd*, *J* = 8.3, 1.8 Hz, ^1^H), 7.50–7.37 ppm (*m*, ^1^H), 7.23–7.13 ppm (*m*, ^1^H), 7.15 ppm (*d*, *J* = 1.8 Hz, ^1^H) and 7.03 ppm (*t*, *J* = 7.1 Hz, ^1^H).

The mass spectroscopy (electron ionization) mass-to-charge ratios (%) were as follows: 542 (M^+^, 100), 381 (13), 353 (16), 324 (33) and 162 (50). The high-resolution mass spectra values were as follows: for C_28_H_14_O_2_Br_2_, calculated, 539.9361; found, 539.9368.

### On-surface synthesis

Low-temperature STM measurements were performed using a commercial Scienta-Omicron LT-STM at 4.3 K. The system consists of a preparation chamber with a typical pressure in the low 10^–10^ mbar regime and an STM chamber with a pressure in the 10^–11^ mbar range. The Au(111) crystal was cleaned via cycles of argon sputtering and annealing (720 K). In order to form pristine (3,1)-chGNRs, a racemic mixture of chiral DBBA precursors was deposited onto the Au(111) surface (held at room temperature) via sublimation at 433 K. Following this, the sample was typically annealed to approximately 600–620 K for 20 minutes in order to form the nanoribbons. To form k-chGNRs, a mixture of *E* and *Z* ketone–DBBA precursors (Supplementary Information for details on the synthesis of these precursors) was deposited onto the room temperature Au(111) crystal via sublimation at 448 K. In order to form flat k-chGNRs, the sample was typically heated to around 670–700 K after deposition. Heating straight to this temperature typically yielded more metal-coordinated GNRs than ‘typical’ GNRs. If the sample is instead heated to a moderately high temperature (for example, 650 K) and then annealed in steps of 15–20 K up to 700 K, then more ‘typical’ GNRs are usually found on the surface. In our case, the sample was cooled in between each step to investigate whether GNRs had been formed, but it is not clear whether this was necessary. More investigation would be required to understand the kinetics of the formation of the different types of k-chGNRs.

### GNR hydrogenation

Hydrogenation of the samples was achieved with a hydrogen cracking source with a leak valve and a narrow inlet that flows through a tungsten tube into the chamber. The H_2_ flow is such that the pressure in the preparation chamber increases to 1 × 10^–7^ mbar, after which the tungsten tube was heated (via electron-beam heating) to around 2,800 K with a heating power of 80 W (corresponding to an acceleration voltage of 1,000 V and 80 mA emission current)—this temperature is high enough to split substantial amounts of the molecular hydrogen into atomic hydrogen, but there is no way of measuring the flux of atomic H on the simple hydrogen source used for these experiments. The sample was then placed in front of the source for 1–2 minutes in the experiments described in the main text. Thereafter, the sample was annealed to around 570–600 K to dehydrogenate the nanoribbons and form p-chGNRs. The lightly hydrogenated samples used for the nc-AFM experiments were obtained by backfilling of the preparation chamber at a pressure of 1 × 10^–6^ mbar of H_2_, maintaining the sample in proximity to (~15 cm) and facing the ion gauge for 1 hour. Control experiments with the ion gauge turned off showed no hydrogenation.

### GNR exposure to ambient conditions

To study the ambient condition exposure, the Au(111) sample with the GNRs was transferred to the fast entry load-lock chamber. Next, that chamber was vented by air at ambient conditions (21 °C, 75% relative humidity). After a 24 minute exposure period, the chamber was pumped again and the sample was transferred back for further STM analysis. To remove most of the contamination that comes along with the air exposure, the sample was annealed to 200 °C before its new STM analysis.

### STM characterization

All STM measurements shown were performed at 4.3 K. The tunnelling current set points and bias voltages used are mentioned in the figure captions for each image. To obtain BR-STM images, the tip was functionalized with a CO molecule that was picked up from the Au(111) surface. To deposit CO, the STM chamber was filled to a pressure of approximately 1 × 10^–8^ mbar of pure CO, and the Au(111) sample was exposed via opening the STM radiation shields. Each deposition was limited in time, such that the temperature of the sample did not exceed 7 K due to radiative heating. Picking up a CO molecule from the surface was achieved via scanning at high tunnelling current set points (1 nA) and negative bias voltages (typically −0.5 V to −1.0 V). A sudden change in apparent tip height and resolution was usually observed upon picking up a CO. BR-STM images were obtained by scanning a CO tip over the molecule at constant height with a low bias voltage (usually 5–8 mV). The d*I*/d*V* measurements were recorded with the internal lock-in of the system. Typical oscillation parameters were 828 Hz and 20 mV.

### The nc-AFM characterization

The nc-AFM experiments were performed in an ultra-high vacuum system with a base pressure below 5 × 10^–10^ mbar, and hosting an STM/nc-AFM instrument (Createc) operated at 4.2 K. The nc-AFM images were taken with Pt/Ir tips that had been sharpened by a focused ion beam and mounted on a qPlus sensor. The tip was functionalized at low temperature with a single CO molecule picked up from the bare metal substrate and operated in frequency-modulation mode (oscillated with a constant amplitude of 50 pm; resonant frequency, ~30 kHz; stiffness, ~1,800 N m^–1^). All nc-AFM images were acquired in constant height mode.

### XPS characterization

XPS was carried out holding the sample at room temperature and illuminating it with monochromatized Al Kα light from a microfocus set-up (SPECS Focus 600). The excited photoelectrons were collected by a SPECS 150 analyser at an emission angle of 40°. The overall experimental resolution was extracted from Fermi edge analysis and resulted in 0.4 eV. The sample was first checked for possible contamination, which was not detected. Then the C 1*s* spectral region was investigated. The spectrum of ‘as-grown’ p-chGNRs can be decently fitted by one single peak (Supplementary Fig. 3a). We therefore refrain from overfitting with additional components as typically performed with higher-resolution synchrotron-radiation-based XPS spectra^45,46^ and instead assign this peak to *sp*^2^ C atoms (labelled as C*sp*^2^). Upon hydrogenation, the energy and full-width at half-maximum of the C*sp*^2^ component is maintained as fixed while leaving the intensity to be fitted, and the spectral broadening towards lower binding energy is accounted for by including a second component that we now associate with hydrogenated *sp*^3^ atoms (labelled as C*sp*^3^), which are indeed expected to appear at lower binding energies^42^. The two fitted components are shifted by only ~0.1 eV, much less than the component’s full-width at half-maximum (~1 eV), and therefore mostly overlap (remember that the C*sp*^2^ component also includes hydrogenated edge atoms). Although this fit can therefore not be considered conclusive, the resulting energy and width for the new *sp*^3^ component coincide with those used for fitting the k-chGNRs, and furthermore, the fit displays a *sp*^3^ to *sp*^2^ ratio of 87:13, in qualitative agreement with estimations based on scanning probe microscopy. Importantly, after annealing the hydrogenated GNRs, the initial spectral shape is recovered, being fitted again only with the *sp*^2^ component and thereby confirming the reversibility of the protection/deprotection process.

The k-chGNR case is slightly more complex. To start with, the initial spectrum of ‘as-grown’ ribbons is less symmetric and is thus fitted with two components in a 1 to 13 intensity ratio for the high and low binding energy signal, respectively. This ratio corresponds to the stoichiometry of the *sp*^2^ carbon atoms bound to the more electronegative oxygen atom (and thus at a higher binding energy; labelled as C=O) and the remaining *sp*^2^ carbon atoms bound only to C or to C and H (and thus at a lower binding energy due to the lower electronegativity of C and H as compared to O; labelled as k-C*sp*^2^). Note that the k-C*sp*^2^ component is at a higher binding energy than the C*sp*^2^ component of the p-chGNRs due to the electron-withdrawing effect of the ketone. As the ribbons are hydrogenated, there is a notable shift of the spectral weight to lower binding energies. In the fit, we account for this change while maintaining the C=O and k-C*sp*^2^ components unchanged (except for their intensity) and adding a new C*sp*^3^ component for the hydrogenated atoms at the same energy as for the p-chGNR case. With these constraints, we observe that the intensity of both the C=O and k-C*sp*^2^ components is reduced to 56% of the initial values, supporting the deoxidation readily at room temperature during the hydrogenation, as previously concluded from the scanning probe microscopy analysis. After annealing, these two components decrease even further to 32% of their initial value, in qualitative agreement with the ‘deprotection’ efficiency estimated from the scanning probe microscopy analysis. The remaining intensity is made up by a C*sp*^2^ component that supports the conversion of the k-chGNRs into p-chGNRs. It is important to note that the total intensity during the hydrogenation/annealing cycle remains virtually unchanged, supporting the absence of any important desorption during the treatment.

### Theoretical calculations

DFT calculations were performed using the all-electron FHI-AIMS code^47^ with the hybrid exchange–correlation functional B3LYP (ref. ^48^) to describe the electronic properties of different GNR models in the gas phase. In all the calculations, we employed the light settings for the numerical atomic basis sets. Slab DFT calculations of different GNR models on Au(111) (Supplementary Fig. 18) were carried out using the generalized gradient approximation Perdew–Burke–Ernzerhof^49^ exchange–correlation functional due to its feasible computational cost compared to the hybrid functional B3LYP. We have checked that Perdew–Burke–Ernzerhof and B3LYP provide a similar character of atomic and electronic structure of free-standing chGNR. In the calculations including the substrate, we employed the Tkatchenko–Scheffler^50^ method to describe van der Waals interactions. We use rectangular slabs comprising four layers of 3 × 8 surface atoms for pristine and ketone-functionalized GNRs and two layers of 3 × 10 surface atoms for Au-coordinated k-chGNRs. The bottom layer was fixed, and the top three layers (one for metal-coordinated islands) and the chGNR atoms, as well as atoms coordinated to the ribbons, were allowed to move without constraints. To keep the optimal lattice vector of GNRs with a periodic epitaxial registry on the Au(111) substrate while maintaining these reduced superstructure unit cells, we extended the Au lattice constant to 2.96 Å and neglected the herringbone reconstruction. In the slab calculations, the chGNRs and k-chGNRs were separated by ~11 Å and the metal-coordinated GNRs in islands by ~3.3 Å. In all total energy calculations, the atomic structures were relaxed until the total forces reached below 10^–2^ eV Å^–1^. For the structural optimization of infinite systems with a one-dimensional periodic boundary condition (pristine and ketone-functionalized GNRs), a Monkhorst–Pack^51^ grid of 18 × 1 × 1 was used to sample the Brillouin zone; and for the Au-coordinated ketone-functionalized ribbons with two-dimensional periodicity, a grid of 18 × 18 × 1 *k* points was used. All band structure plots presented here were obtained with 50 *k* points along the one-dimensional periodicity direction. Theoretical d*I*/d*V* maps were calculated by the DFT Fireball^52^ program package and Probe Particle STM (PP-STM) code^53,54^ for a CO-like orbital tip, represented by 95% *p*_*x*_*p*_*y*_-wave and 5% *s*-wave character.

## Online content

Any methods, additional references, Nature Research reporting summaries, source data, extended data, supplementary information, acknowledgements, peer review information; details of author contributions and competing interests; and statements of data and code availability are available at 10.1038/s41557-022-01042-8.

### Supplementary information

Supplementary Information Supplementary Figs. 1–18.

Supplementary Data 1 Source data for Supplementary Fig. 1.

Supplementary Data 2 Source data for Supplementary Fig. 9.

### Source data

Source Data Fig. 2 Scanning probe microscopy images for Fig. 2.

Source Data Fig. 3 Scanning probe microscopy images for Fig. 3.

Source Data Fig. 4 Conductance spectra, scanning probe microscopy images, calculated band structure and calculated wavefunctions for Fig. 4.

Source Data Fig. 5 Conductance spectra and calculated atomic positions for Fig. 5

Source Data Fig. 6 Scanning probe microscopy images for Fig. 6.

Source Data Extended Data Fig. 1 Unprocessed XPS data for the pristine and ketone-functionalized ribbons as grown, after hydrogen exposure and after subsequent annealing.

Source Data Extended Data Fig. 2 Scanning probe microscopy images for Extended Data Fig. 2.

Source Data Extended Data Fig. 3 Scanning probe microscopy images for Extended Data Fig. 3.

## Data Availability

All relevant data generated and analysed during this study, including STM and scanning tunnelling spectroscopy data and theoretical calculations, are included in this Article and its Supplementary Information and are also available from the authors upon reasonable request. Source data are provided with this paper.
