# Publisher Correction: Circumventing the stability problems of graphene nanoribbon zigzag edges

**DOI:** 10.1038/s41557-022-01105-w

**Published:** 2022-12-14

**Authors:** James Lawrence, Alejandro Berdonces-Layunta, Shayan Edalatmanesh, Jesús Castro-Esteban, Tao Wang, Alejandro Jimenez-Martin, Bruno de la Torre, Rodrigo Castrillo-Bodero, Paula Angulo-Portugal, Mohammed S. G. Mohammed, Adam Matěj, Manuel Vilas-Varela, Frederik Schiller, Martina Corso, Pavel Jelinek, Diego Peña, Dimas G. de Oteyza

**Affiliations:** 1 Donostia International Physics Center, San Sebastián, Spain; 2 Centro de Física de Materiales (MPC), CSIC-UPV/EHU, San Sebastián, Spain; 3 Institute of Physics, Czech Academy of Sciences, Prague, Czech Republic; 4 Centro Singular de Investigación en Química Biolóxica e Materiais Moleculares (CiQUS) and Departamento de Química Orgánica, Universidade de Santiago de Compostela, Santiago de Compostela, Spain; 5 Regional Centre of Advanced Technologies and Materials, Czech Advanced Technology and Research Institute (CATRIN), Palacký University Olomouc, Olomouc, Czech Republic; 6 Faculty of Nuclear Sciences and Physical Engineering, Czech Technical University in Prague, Prague, Czech Republic; 7 Ikerbasque, Basque Foundation for Science, Bilbao, Spain; 8 Nanomaterials and Nanotechnology Research Center (CINN), CSIC-UNIOVI-PA, El Entrego, Spain

**Keywords:** Electronic materials, Synthesis and processing, Electronic properties and materials, Scanning probe microscopy

Correction to: *Nature Chemistry* 10.1038/s41557-022-01042-8, published online 26 September 2022.

In the version of this article initially published, a few double bonds were missing in Figs. 3a, 5a and 6i. Original and corrected panels are shown below (Figs. 1–4), and the HTML and PDF versions of the article have been updated.

**Fig. 1:**
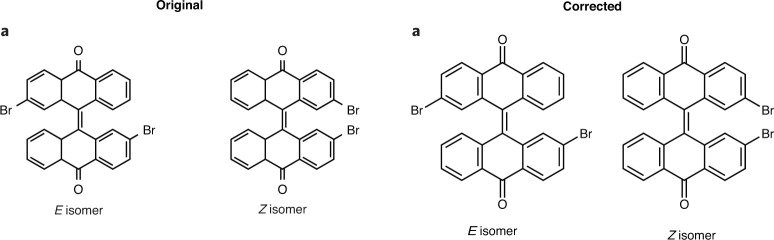
Original and corrected Figure 3a.

**Fig. 2:**
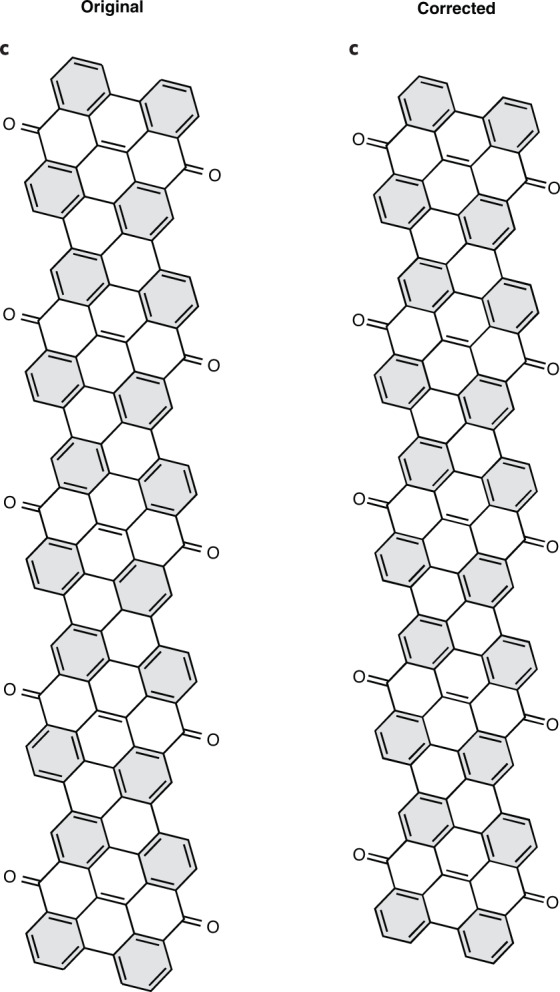
Original and corrected Figure 3c.

**Fig. 3:**
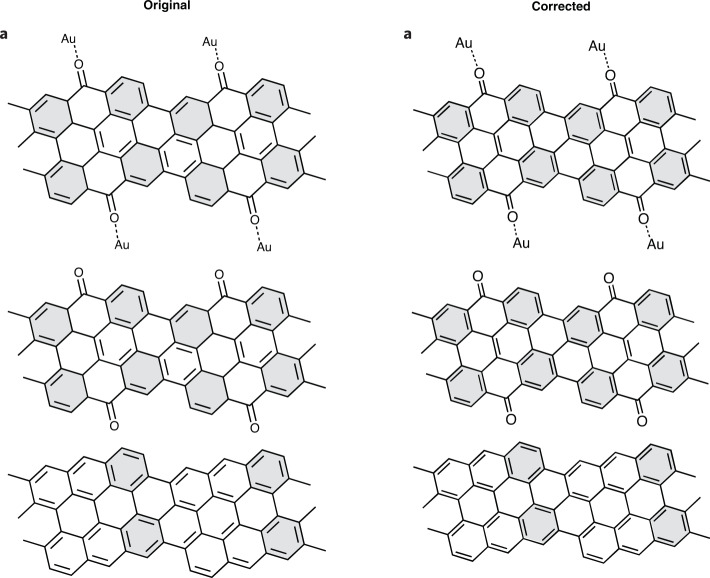
Original and corrected Figure 5a.

**Fig. 4:**
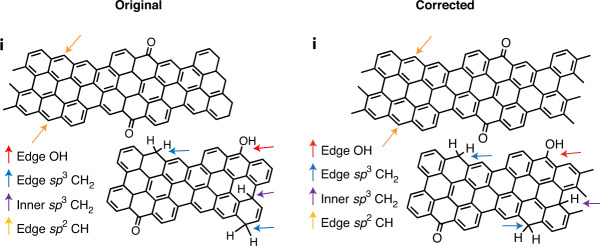
Original and corrected Figure 6i.

