# Author Correction: Circumventing the stability problems of graphene nanoribbon zigzag edges

**DOI:** 10.1038/s41557-023-01324-9

**Published:** 2023-11-22

**Authors:** James Lawrence, Alejandro Berdonces-Layunta, Shayan Edalatmanesh, Jesús Castro-Esteban, Tao Wang, Alejandro Jimenez-Martin, Bruno de la Torre, Rodrigo Castrillo-Bodero, Paula Angulo-Portugal, Mohammed S. G. Mohammed, Adam Matěj, Manuel Vilas-Varela, Frederik Schiller, Martina Corso, Pavel Jelinek, Diego Peña, Dimas G. de Oteyza

**Affiliations:** 1 Donostia International Physics Center, San Sebastián, Spain; 2 Centro de Física de Materiales (MPC), CSIC-UPV/EHU, San Sebastián, Spain; 3 Institute of Physics, Czech Academy of Sciences, Prague, Czech Republic; 4 Centro Singular de Investigación en Química Biolóxica e Materiais Moleculares (CiQUS) and Departamento de Química Orgánica, Universidade de Santiago de Compostela, Santiago de Compostela, Spain; 5 Regional Centre of Advanced Technologies and Materials, Czech Advanced Technology and Research Institute (CATRIN), Palacký University Olomouc, Olomouc, Czech Republic; 6 Faculty of Nuclear Sciences and Physical Engineering, Czech Technical University in Prague, Prague, Czech Republic; 7 Ikerbasque, Basque Foundation for Science, Bilbao, Spain; 8 Nanomaterials and Nanotechnology Research Center (CINN), CSIC-UNIOVI-PA, El Entrego, Spain

**Keywords:** Electronic materials, Synthesis and processing, Electronic properties and materials, Scanning probe microscopy

Correction to: *Nature Chemistry* 10.1038/s41557-022-01042-8. Published online 26 September 2022.

This paper was originally published under a standard Springer Nature license (© The Author(s), under exclusive licence to Springer Nature Limited). It is now available as an Open Access paper under a Creative Commons Attribution 4.0 International license, © The Author(s). The error has been corrected in the HTML and PDF versions of the article.

