# Supplementary material for: Circumventing the stability problems of graphene nanoribbon zigzag edges
Source: Nat Chem. 2022 Sep 26;14(12):1451–8. doi: 10.1038/s41557-022-01042-8 (PMC10665199; doi:10.1038/s41557-022-01042-8)
Supplement: Supplementary file 1 — Supplementary Figs. 1–18. [file 41557_2022_1042_MOESM1_ESM.pdf]

nature portfolio

https://doi.org/10.1038/s41557-022-01042-8

Supplementary information

Circumventing the stability problems of graphene nanoribbon zigzag edges

In the format provided by the authors and unedited

Supplementary Information for

Circumventing the Stability Problems of Graphene Nanoribbon Zigzag Edges

James Lawrence[1,2,†], Alejandro Berdonces-Layunta[1,2,†], Shayan Edalatmanesh,[3,5] Jesús Castro-Esteban,[4] Tao Wang[1,2], Alejandro Jimenez-Martin,[3,5,6] Bruno de la Torre,[3,5] Rodrigo Castrillo-Bodero,[2] Paula Angulo-Portugal,[2] Mohammed S. G. Mohammed,[1,2] Adam Matěj,[3,5] Manuel Vilas-Varela,[4] Frederik Schiller,[1,2] Martina Corso,[1,2] Pavel Jelinek,[3,5*] Diego Peña[4,*], Dimas G. de Oteyza[1,2,7,8,*]

[1]Donostia International Physics Center; 20018 San Sebastián, Spain

[2] Centro de Física de Materiales (MPC), CSIC-UPV/EHU; 20018 San Sebastián, Spain

[3]Institute of Physics, Czech Academy of Sciences; 16200 Prague, Czech Republic

[4]Centro Singular de Investigación en Química Biolóxica e Materiais Moleculares (CiQUS) and Departamento de Química Orgánica, Universidade de Santiago de Compostela; 15782 Santiago de Compostela, Spain

[5]Regional Centre of Advanced Technologies and Materials, Czech Advanced Technology and Research Institute (CATRIN), Palacký University Olomouc, 78371 Olomouc, Czech Republic.

[6] Faculty of Nuclear Sciences and Physical Engineering, Czech Technical University in Prague, Brehova 7, 115 19 Prague 1, Czech Republic

[7] Ikerbasque, Basque Foundation for Science; 48013 Bilbao, Spain

[8] Nanomaterials and Nanotechnology Research Center (CINN), CSIC-UNIOVI-PA; 33940 El Entrego, Spain

* Corresponding author. Email: jelinekp@fzu.cz; diego.pena@usc.es; d.g.oteyza@cinn.es

† These authors contributed equally to this work

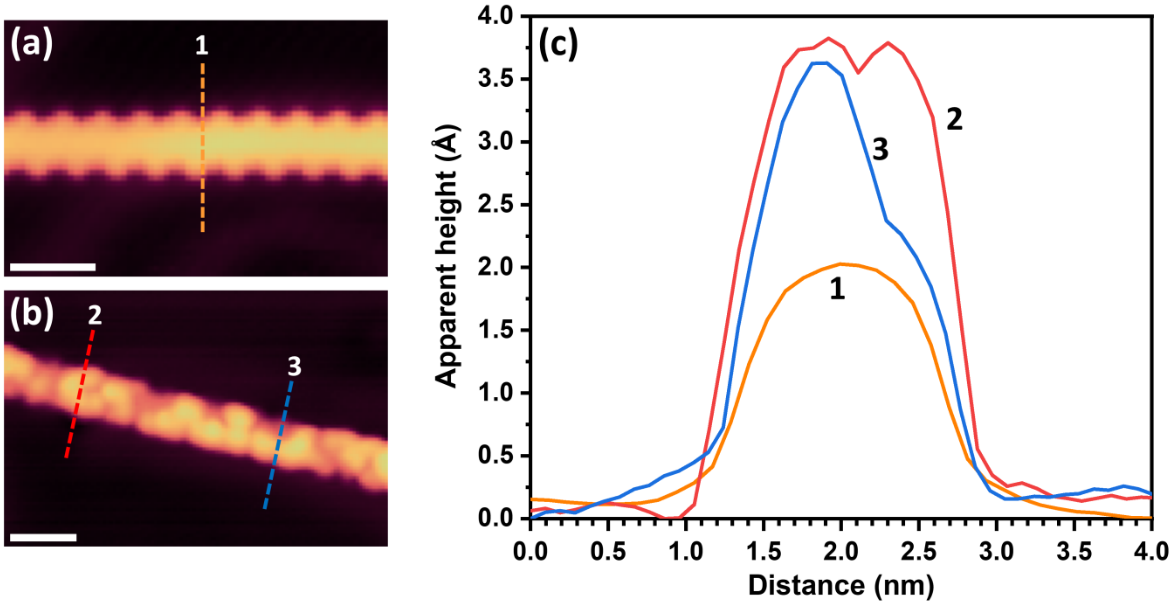

Supplementary Fig. 1. Comparative height profiles of constant current STM images on pristine and hydrogenated GNRs.

STM images of (a) pristine and (b) hydrogenated chiral nanoribbons ((3,1)-chGNRs). (a) I = 1.5 nA; U = −0.5 V. (b) I = 23 pA; U = −2.0 V. Scale bars are both 2 nm. (c) Relative apparent height profiles of the lines shown in (a) and (b). The hydrogenated nanoribbons appear to be approximately twice as high as the pristine nanoribbons; the darker sections are a similar height to the pristine GNRs, which suggests that they are not hydrogenated to the same extent.

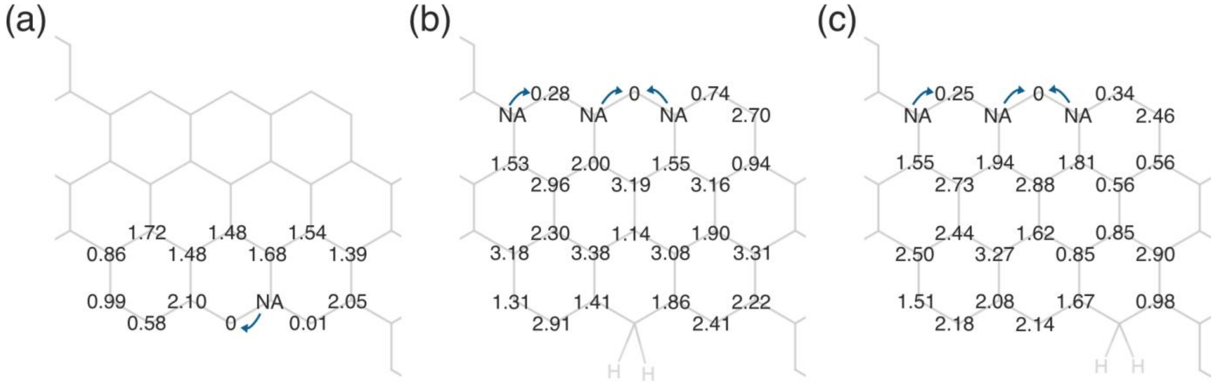

Supplementary Fig. 2. **Total energy differences (in eV) for different steps of hydrogenation calculated as difference from the most stable position.**
(a) Addition of first atomic H on pristine chGNR. (b-c) Second addition after first H added to middle and corner zig-zag edge, respectively. Positions with NA were not stable, and hydrogens migrated to positions depicted by blue arrows. The most favourable sites for hydrogenation are the central atoms on the zigzag segments.

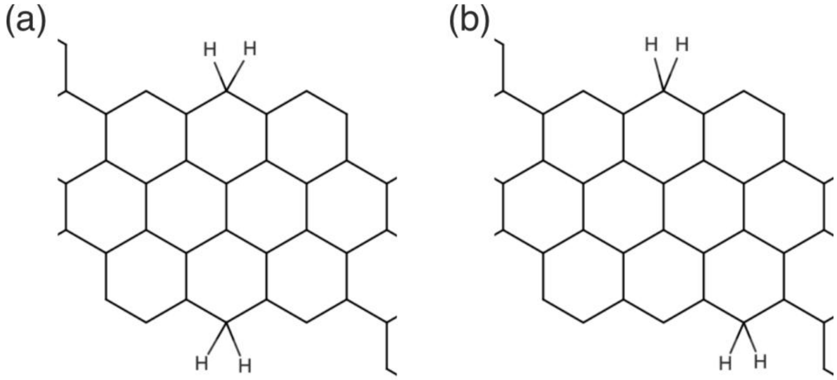

Supplementary Fig. 3. **Total energy difference of two possible doubly hydrogenated structures differing in first step of hydrogenation.**
(a) Reference energy for the lowest energy structure after double hydrogenation of a unit cell. (b) Most favourable structure of a doubly hydrogenated unit cell after an initial hydrogenation on the second most favourable edge atom, revealing the energetic preference for hydrogenating the two central carbon atoms of the zigzag segments on either side of the ribbon as displayed in panel (a).

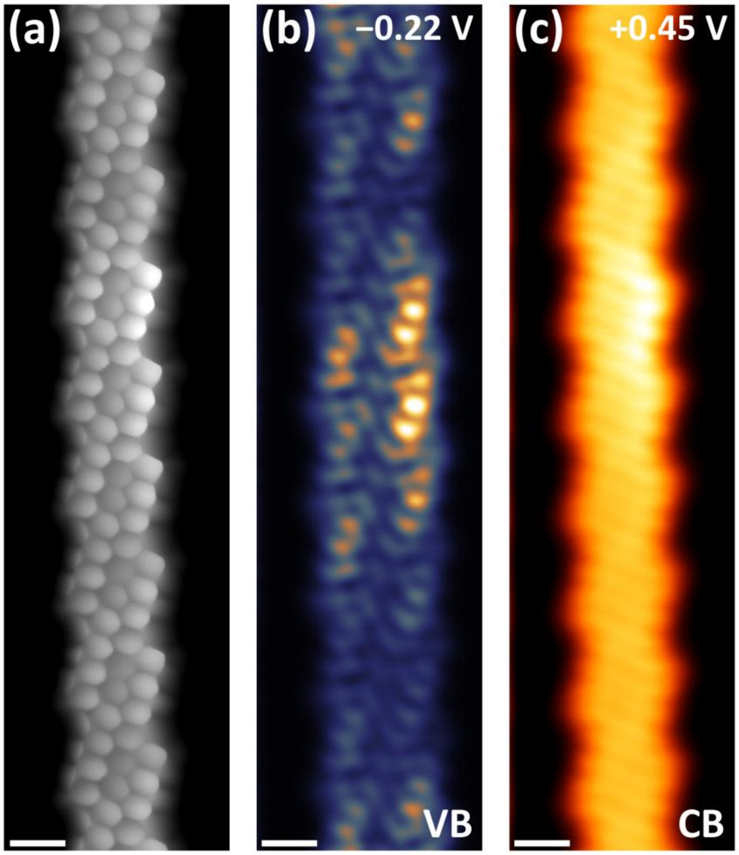

Supplementary Fig. 4. Structural and electronic characterization of GNRs after a protection/deprotection cycle.

(a) BR-STM image of a pristine GNR section after hydrogenation, air exposure and post-annealing to 300°C. Constant height, CO tip, V_{bias} = 5 mV. (b) and (c) Constant height dI/dV images (CO tip) of the same pristine GNR section, demonstrating that the features of the valence and conduction bands are preserved after these treatments. The brighter regions of the images are due to the underlying herringbone reconstruction of the Au(111). Scale bars: 500 pm.

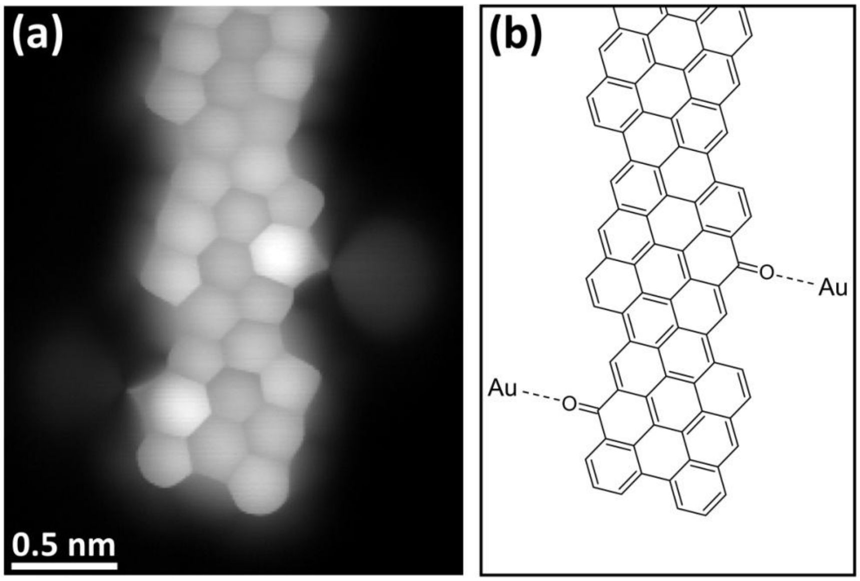

Supplementary Fig. 5. Representative structure of a remaining defect after a GNR deprotection.

(a) BR-STM image and (b) chemical model of a pair of ketone-metal defects at one end of a (mostly) pristine chiral nanoribbon. Constant height, CO tip, V_{bias} = 5 mV. These defects are found in various different configurations (alone, in pairs on the same GNR section, etc.). This example is from the ketone GNR sample that was hydrogenated and then annealed. This implies that not all of the oxygens are removed from the GNRs during the hydrogenation/annealing process. The same defects were often observed with the pristine GNR sample that was hydrogenated, exposed to air and annealed. It follows that these defects probably occur on positions that were not hydrogenated, and thus had reacted with oxygen during the air exposure.

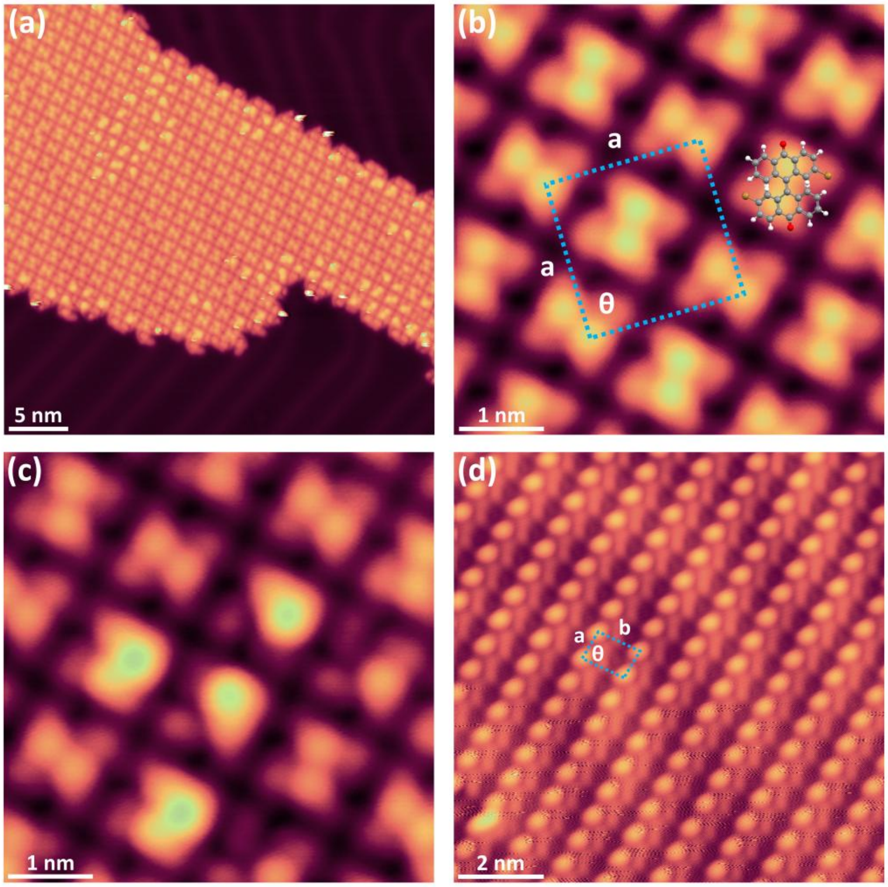

Supplementary Fig. 6. Self-assembly of as-deposited ketone-functionalized precursor molecules.

(a) STM image of an island of ketone-GNR precursors at a relatively low molecular coverage. (b) Zoom of the same island, showing the regular structure and its unit cell. **a** = 1.87 ± 0.03 nm. θ = 90 ± 1°. A scaled gas-phase optimised model of a trans precursor molecule is overlaid in one of its possible conformations for reference. (c) Zoom of a region of an island with 'defects' – possibly different conformations of molecules or a mixture of enantiomers. (d) STM image of a packing that was typically observed at higher coverages. **a** = 0.67 ± 0.02 nm; **b** = 1.06 ± 0.01 nm; θ = 85 ± 2°. All four STM images in this figure were recorded with the following parameters: I = 50 pA; V_{bias} = −0.5 V.

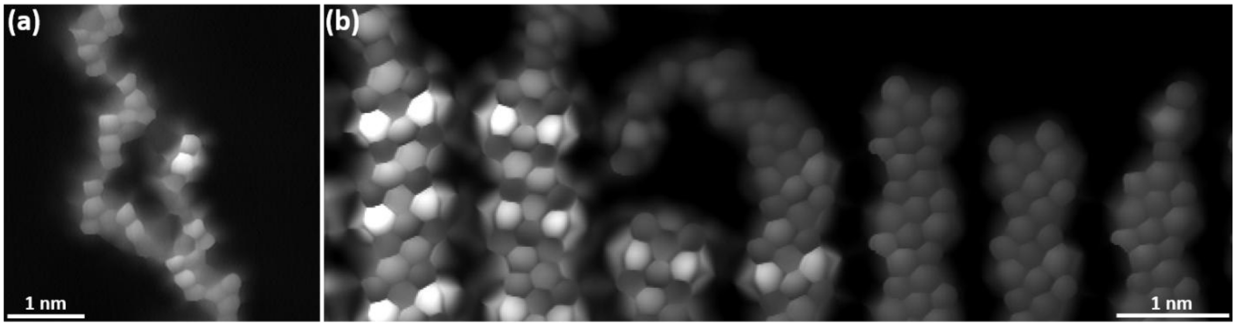

Supplementary Fig. 7. Anthrone-related fragments at ribbon's ends.

(a) BR-STM image (Constant height, CO tip, 5 mV) of a covalently bonded/fused cluster of anthrone-related fragments. (b) BR-STM image (Constant height, CO tip, 5 mV) of the termini of several ketone-GNRs, showing how they are often found fused with an anthrone-related fragment.

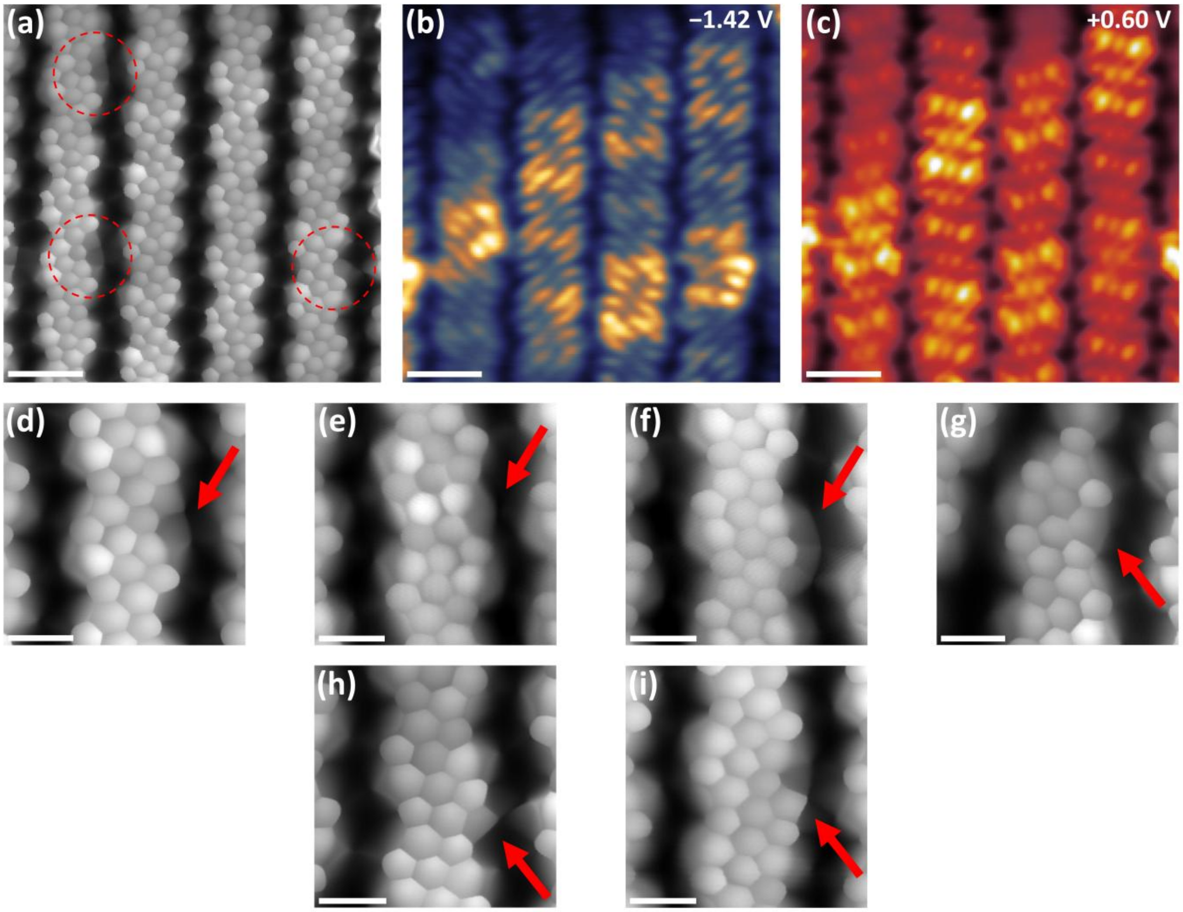

Supplementary Fig. 8. STM analysis of air-exposed k-chGNRs with some representative defect structures.

(a) BR-STM image (Constant height, CO tip, V_{bias} = 5 mV) of an island of ketone-GNRs that were exposed to the atmosphere (24 mins) and post-annealed to 200°C for 1 hour. Defects are highlighted with dashed red circles. (b) and (c) Constant height dI/dV images of the same area (CO tip) at the approximate energies of the valence and conduction bands, respectively. While there are clear changes at the position of the defects, the general spatial distribution of the states is maintained. The appearance of the conduction band onset is relatively unaffected by the defects. Scale bars in (a) – (c) are 1 nm. (d) – (i) BR-STM images (Constant height, CO tip, V_{bias} = 5-8 mV) of various defects that are found after exposing the ketone-GNRs to ambient conditions (plus post-annealing). Many oxidation defects may be found in positions that already possessed defects prior to air exposure, e.g. sections in which ketone groups were missing. This would cause them to be more reactive and readily oxidise. Scale bars in (d) – (i) are 0.5 nm.

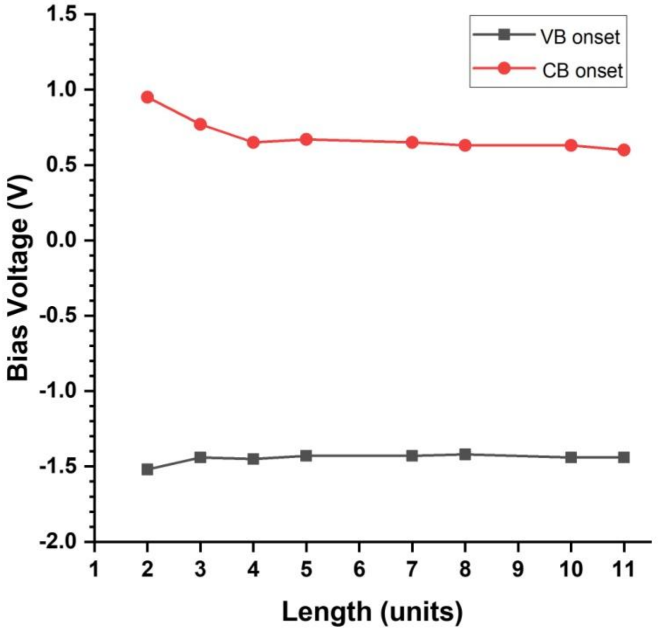

Supplementary Fig. 9. Valence and conduction band onset energies as a function of k-chGNR length.

Dependence of the VB and CB onset positions on the length of the ketone-GNRs. The band gap very quickly saturates after 4 or 5 units in length to a value between 2.04 eV and 2.10 eV, with the smallest gap recorded for the 11-mer.

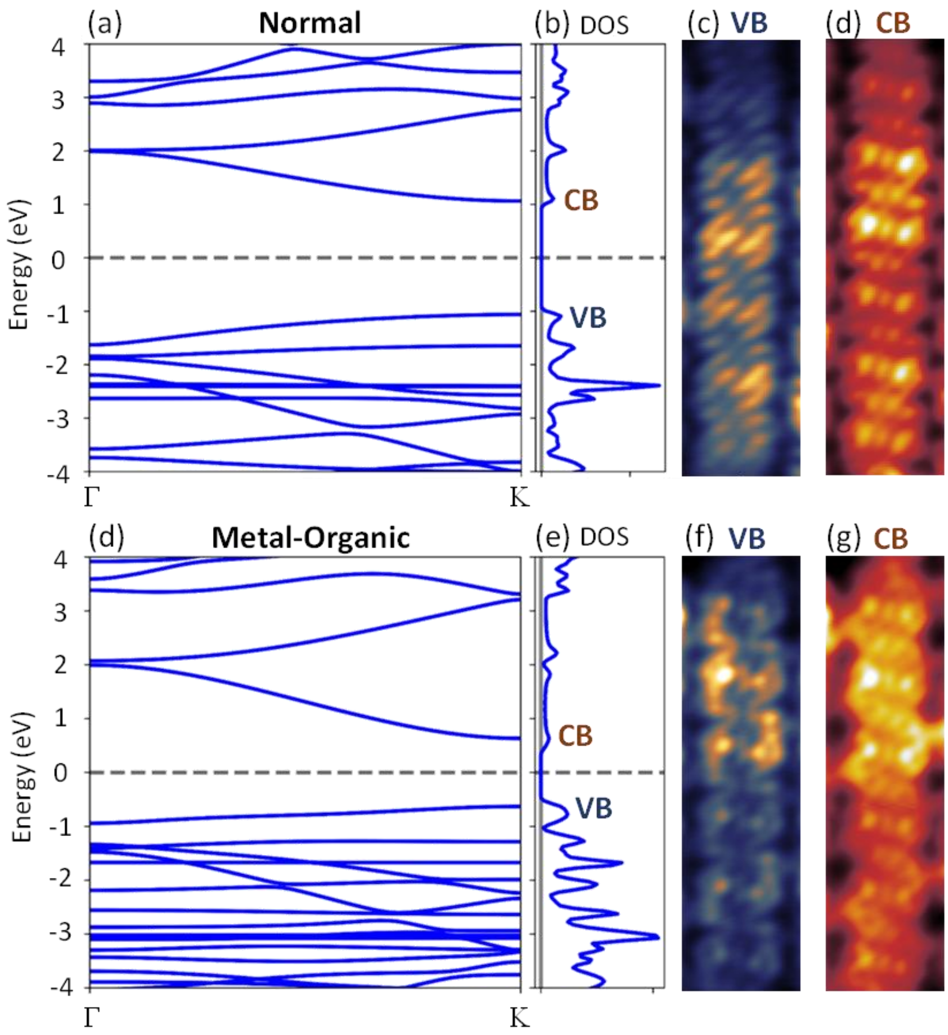

Supplementary Fig. 10. Comparative band structures of typical and metal-coordinated k-chGNRs.

(a) Calculated band structure, (b) integrated DOS, and experimental conductance maps at the (c) VB (U = -1.4 eV) and (d) CB onsets (U = 0.6 eV) of 'typical' k-chGNRs. (d) Calculated band structure, (e) integrated DOS, and experimental conductance maps at the (f) VB (U = -1.6 eV) and (g) CB onsets (U = 0.15 eV) of 'metal-coordinated' k-chGNRs.

The change in the electron conjugation of k-chGNRs upon coordination to Au adatoms is also reflected in the conductance maps. While the general appearance of conductance maps at the CB onset is similar to that of the 'typical' k-chGNRs (**Fig. 13c,f**), at the VB onset the DOS distribution changes substantially (**Fig. S13d,g**). This is again confirmed with DFT simulations. The calculated band structure reveals that the Au-coordination does not affect the CB substantially. However, the VB becomes very flat upon Au-coordination (**Fig. S13a,d**). As a consequence, the conductance maps at the energy of the VB onset now mix contributions from the VB at the K point with previously absent contributions of the VB at lower k values all the way to the Γ point, which are intrinsically favoured by the tunnelling process[1] and display a different spatial distribution.

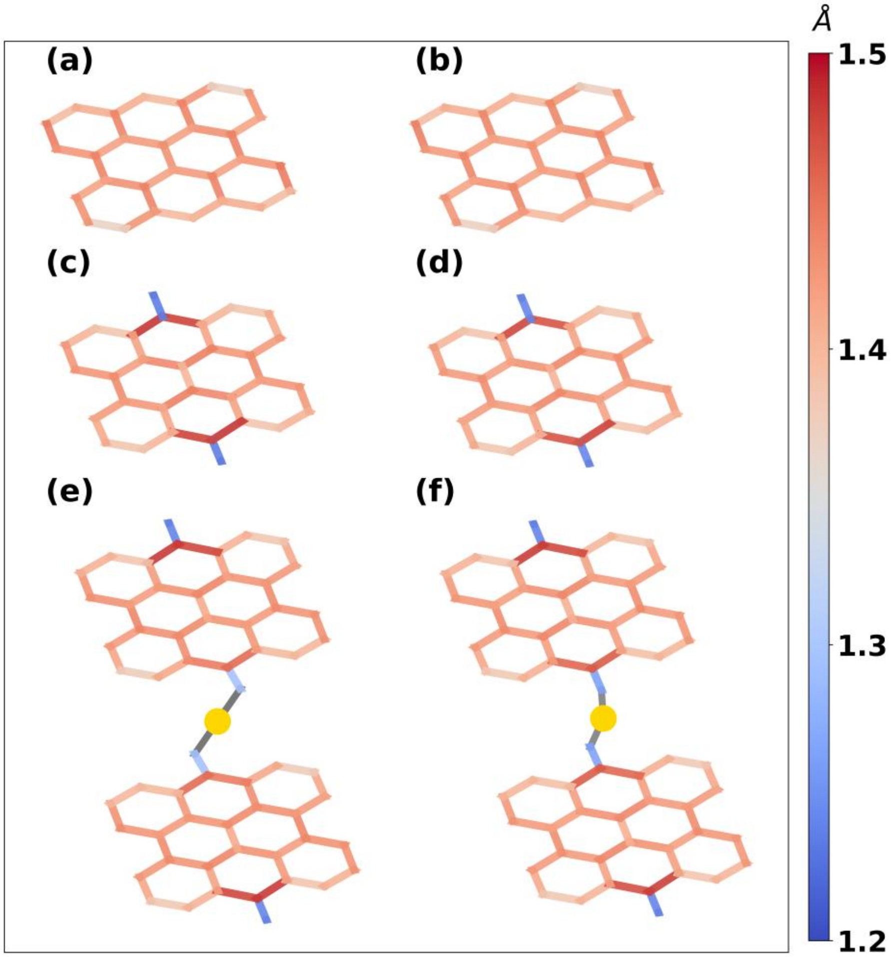

Supplementary Fig. 11. Bond length analysis comparing freestanding and adsorbed ribbons.

Bond length analysis results from geometrically optimized unit cells of: (a) Freestanding p-chGNRs, (b) p-chGNRs on the Au(111) substrate, (c) freestanding k-chGNRs, (d) k-chGNRs on the Au(111) substrate, (e) freestanding metal-coordinated k-chGNR islands, (f) metal-coordinated k-chGNR islands on the Au(111) substrate. The bonds between the Au adatoms and the O atoms are plotted in gray. Panels (e) and (f) represent two different inter-ribbon configurations observed experimentally that, as shown in the next figure, do not affect the bond lengths.

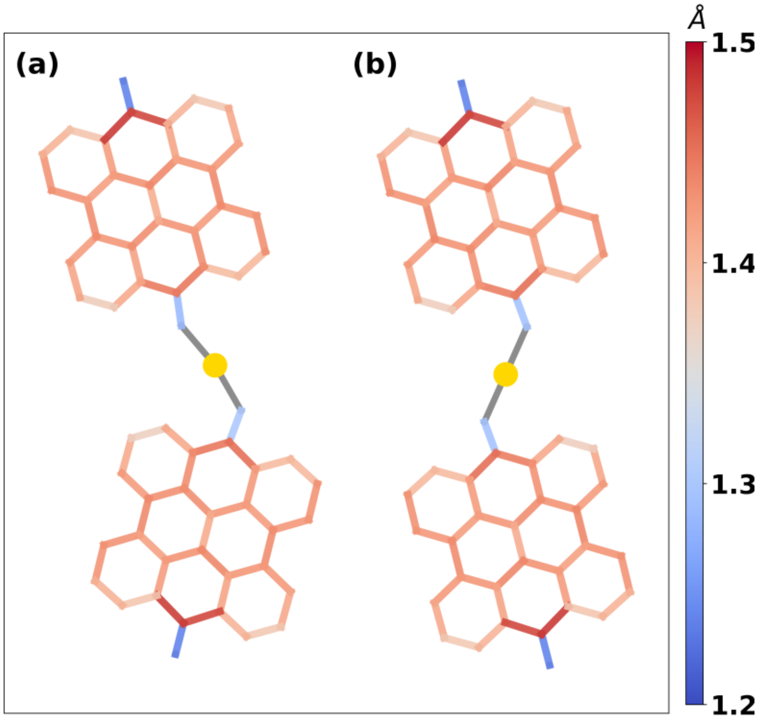

Supplementary Fig. 12. Bond length analysis comparing the two inter-ribbon configurations observed experimentally.

Bond length analysis results from geometrically optimized unit cells of: (a,b) Freestanding metal-coordinated k-chGNR islands in the two different experimentally observed configurations. The alignment of the GNRs doesn't change the bond character.

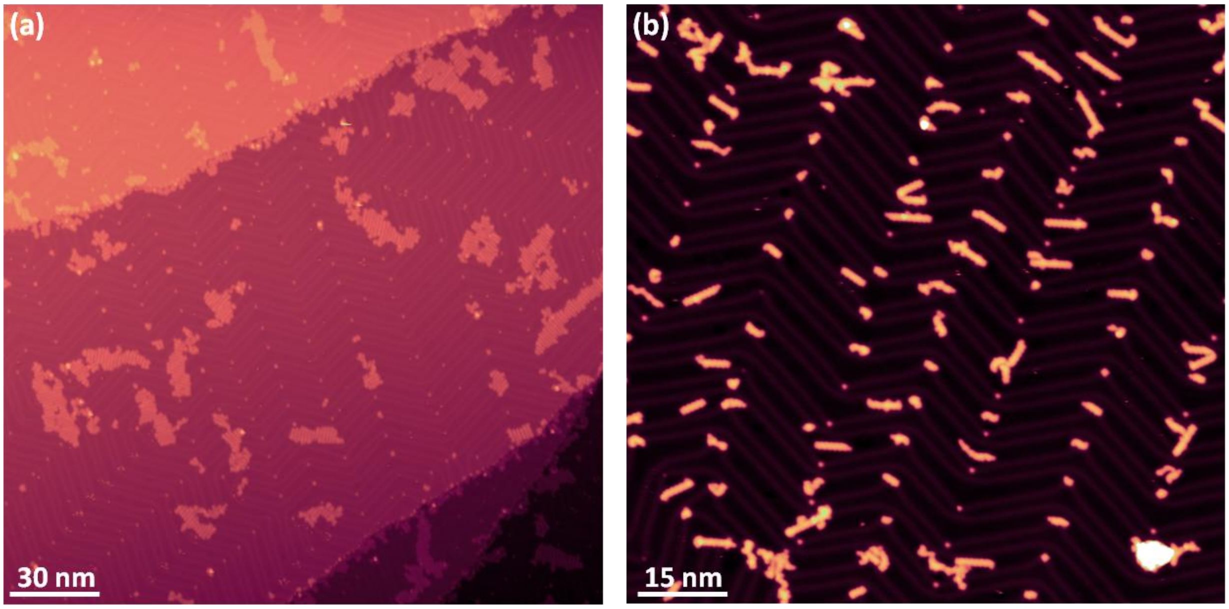

Supplementary Fig. 13. Large scale STM images for coverage assessment.
Large-scale images of the ketone GNRs sample shown in Fig. 6 that show the general coverage of the nanoribbons (approx. 0.1 ML) (a) before and (b) after the hydrogen exposure and annealing. The ketone GNRs before the hydrogenation and annealing process are much less evenly distributed over the surface than the pristine chGNRs due to the formation of self-assembled islands, so a larger image is necessary to properly demonstrate the overall surface coverage.

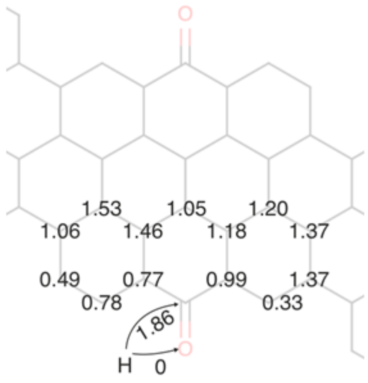

Supplementary Fig. 14. **Total energy differences between different sites for the initial hydrogenation of k-chGNRs.**
Total energy differences (in eV) for addition of atomic H to different positions of k-chGNR, calculated as the difference from the most stable position.

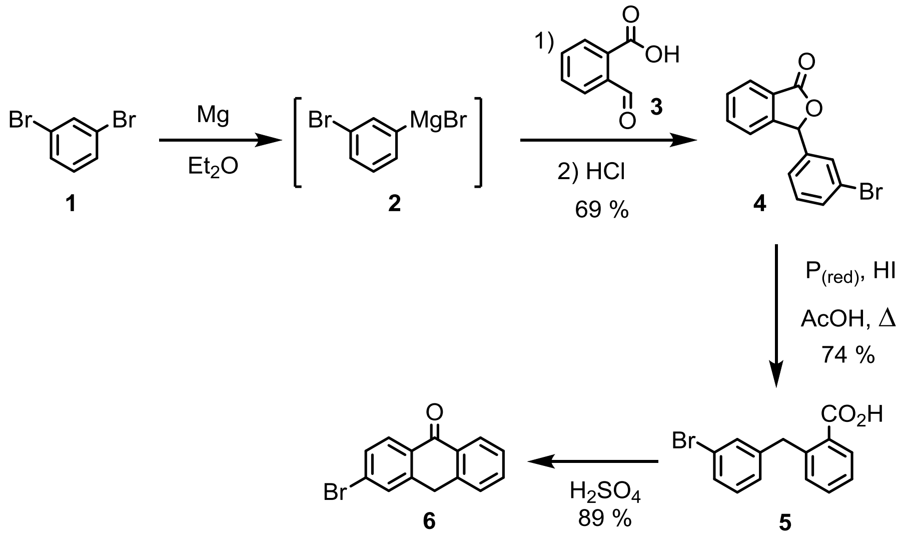

Supplementary Fig. 15. Synthesis of anthrone 6.

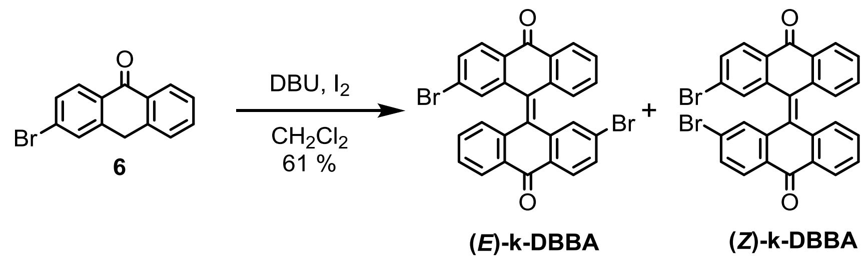

Supplementary Fig. 16. Synthesis of (E/Z)-k-DBBA.

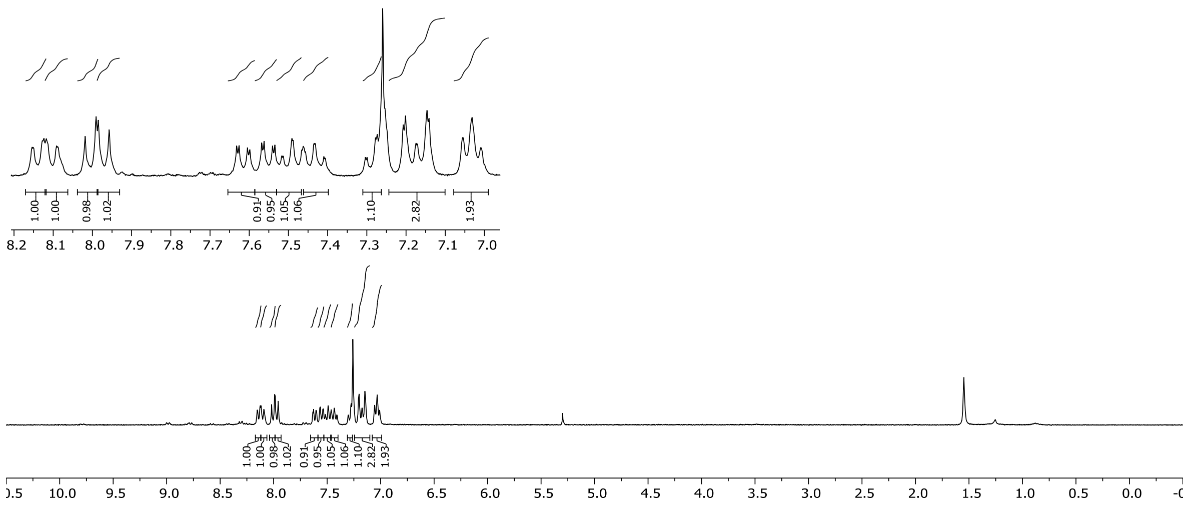

Supplementary Fig. 17. ^{1}H NMR spectra of (E/Z)-k-DBBA.

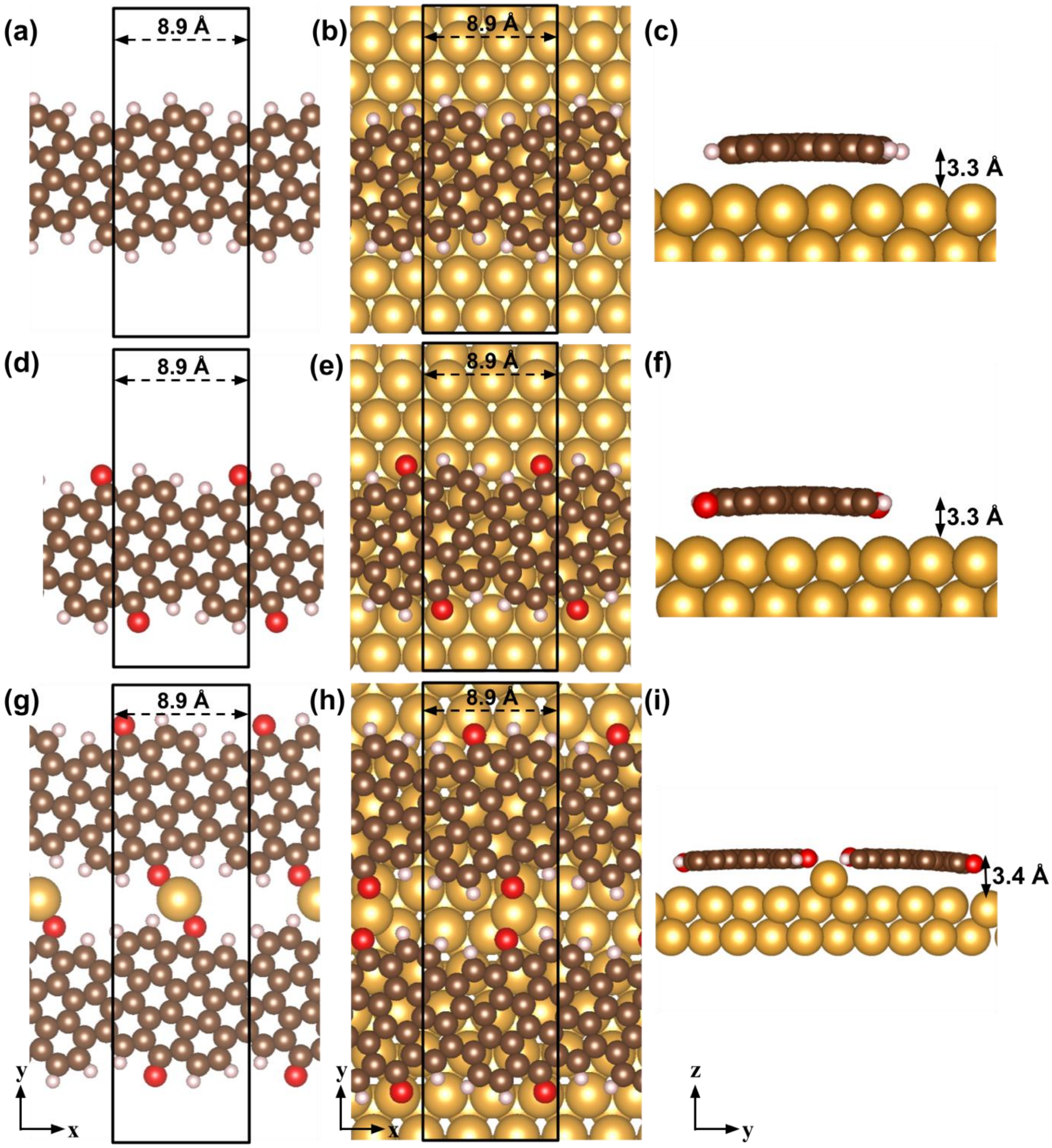

Supplementary Fig. 18. The relaxed models used in DFT calculations.
(a) Pristine chGNRs in gas phase, (b,c) top and side views of the pristine chGNRs on the Au(111) substrate, (d) k-chGNRs in gas phase, (e,f) top and side views of the k-chGNRs on the Au(111) substrate, (g) metal-coordinated k-chGNR islands in gas phase, (h,i) top and side views of the metal-coordinated k-chGNR islands on the Au(111) substrate. The boxes contain the unit cells.

References

1. Chen, J. C. *Introduction to scanning tunneling microscopy*. (Oxford University Press, 2021).
