# Supplementary figures and images for: Circumventing the stability problems of graphene nanoribbon zigzag edges
Source: Nat Chem. 2022 Sep 26;14(12):1451–8. doi: 10.1038/s41557-022-01042-8 (PMC10665199; doi:10.1038/s41557-022-01042-8)

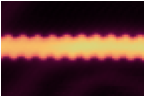

Supplement: Supplementary file 2 — Source data for Supplementary Fig. 1. [file 41557_2022_1042_MOESM2_ESM.zip › FigS1a.png]

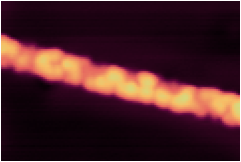

Supplement: Supplementary file 2 — Source data for Supplementary Fig. 1. [file 41557_2022_1042_MOESM2_ESM.zip › FigS1b.png]

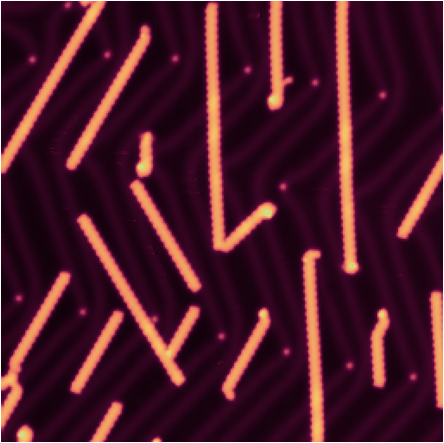

Supplement: Supplementary file 4 — Scanning probe microscopy images for Fig. 2. [file 41557_2022_1042_MOESM4_ESM.zip › Fig2a.png]

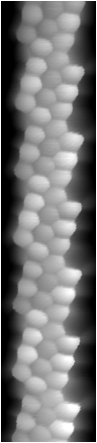

Supplement: Supplementary file 4 — Scanning probe microscopy images for Fig. 2. [file 41557_2022_1042_MOESM4_ESM.zip › Fig2b.png]

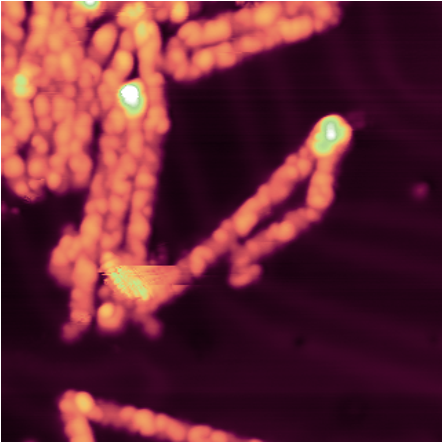

Supplement: Supplementary file 4 — Scanning probe microscopy images for Fig. 2. [file 41557_2022_1042_MOESM4_ESM.zip › Fig2c.png]

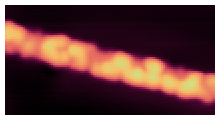

Supplement: Supplementary file 4 — Scanning probe microscopy images for Fig. 2. [file 41557_2022_1042_MOESM4_ESM.zip › Fig2c_inset.png]

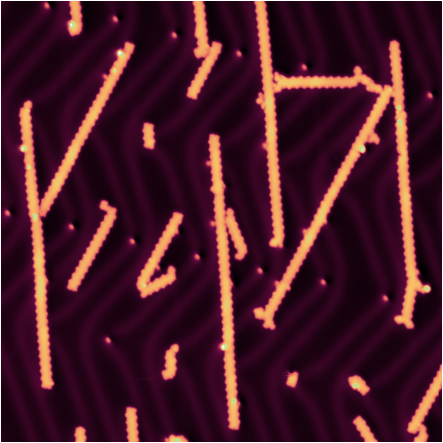

Supplement: Supplementary file 4 — Scanning probe microscopy images for Fig. 2. [file 41557_2022_1042_MOESM4_ESM.zip › Fig2d.png]

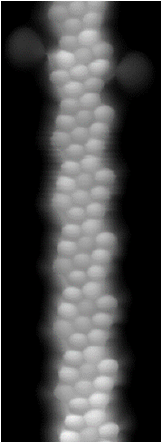

Supplement: Supplementary file 4 — Scanning probe microscopy images for Fig. 2. [file 41557_2022_1042_MOESM4_ESM.zip › Fig2e.png]

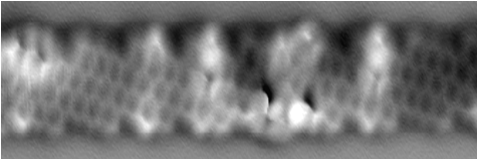

Supplement: Supplementary file 4 — Scanning probe microscopy images for Fig. 2. [file 41557_2022_1042_MOESM4_ESM.zip › Fig2f.png]

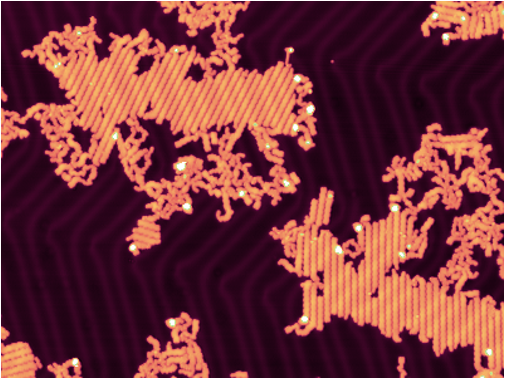

Supplement: Supplementary file 5 — Scanning probe microscopy images for Fig. 3. [file 41557_2022_1042_MOESM5_ESM.zip › Fig3b.png]

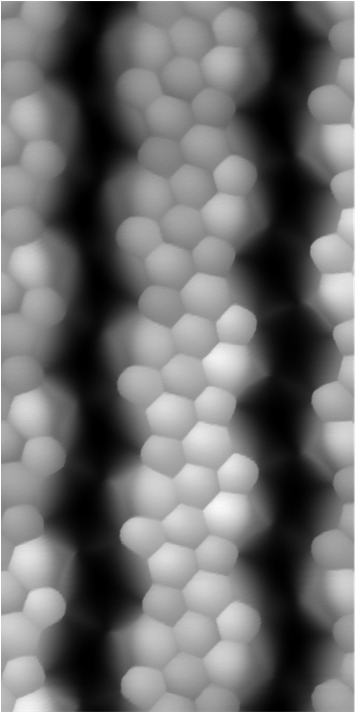

Supplement: Supplementary file 5 — Scanning probe microscopy images for Fig. 3. [file 41557_2022_1042_MOESM5_ESM.zip › Fig3d.png]

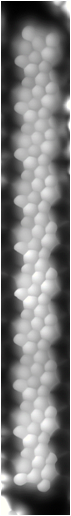

Supplement: Supplementary file 6 — Conductance spectra, scanning probe microscopy images, calculated band structure and calculated wavefunctions for Fig. 4. [file 41557_2022_1042_MOESM6_ESM.zip › Fig4b.png]

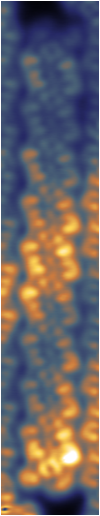

Supplement: Supplementary file 6 — Conductance spectra, scanning probe microscopy images, calculated band structure and calculated wavefunctions for Fig. 4. [file 41557_2022_1042_MOESM6_ESM.zip › Fig4c.png]

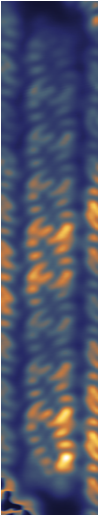

Supplement: Supplementary file 6 — Conductance spectra, scanning probe microscopy images, calculated band structure and calculated wavefunctions for Fig. 4. [file 41557_2022_1042_MOESM6_ESM.zip › Fig4d.png]

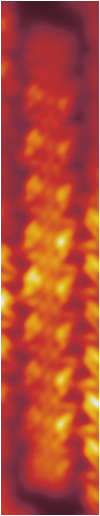

Supplement: Supplementary file 6 — Conductance spectra, scanning probe microscopy images, calculated band structure and calculated wavefunctions for Fig. 4. [file 41557_2022_1042_MOESM6_ESM.zip › Fig4e.png]

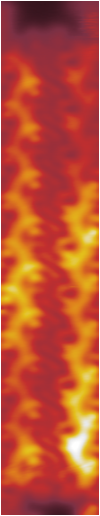

Supplement: Supplementary file 6 — Conductance spectra, scanning probe microscopy images, calculated band structure and calculated wavefunctions for Fig. 4. [file 41557_2022_1042_MOESM6_ESM.zip › Fig4f.png]

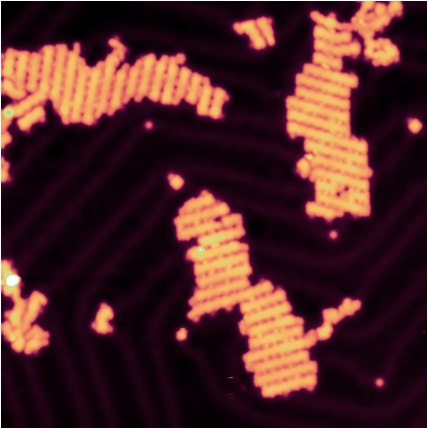

Supplement: Supplementary file 8 — Scanning probe microscopy images for Fig. 6. [file 41557_2022_1042_MOESM8_ESM.zip › Fig6a.png]

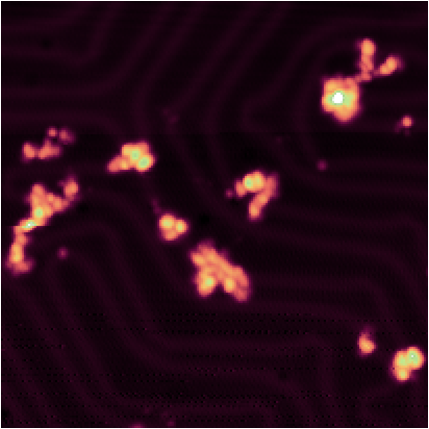

Supplement: Supplementary file 8 — Scanning probe microscopy images for Fig. 6. [file 41557_2022_1042_MOESM8_ESM.zip › Fig6b.png]

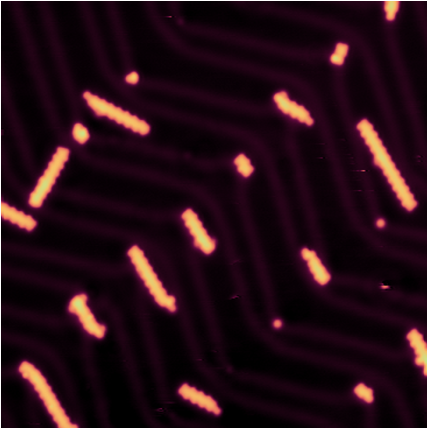

Supplement: Supplementary file 8 — Scanning probe microscopy images for Fig. 6. [file 41557_2022_1042_MOESM8_ESM.zip › Fig6c.png]

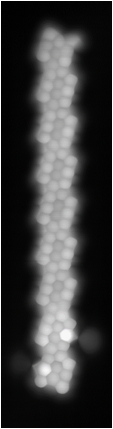

Supplement: Supplementary file 8 — Scanning probe microscopy images for Fig. 6. [file 41557_2022_1042_MOESM8_ESM.zip › Fig6d.png]

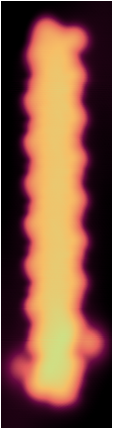

Supplement: Supplementary file 8 — Scanning probe microscopy images for Fig. 6. [file 41557_2022_1042_MOESM8_ESM.zip › Fig6e.png]

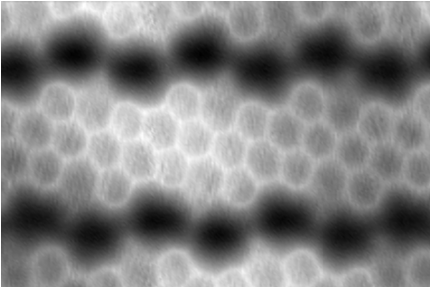

Supplement: Supplementary file 8 — Scanning probe microscopy images for Fig. 6. [file 41557_2022_1042_MOESM8_ESM.zip › Fig6f.png]

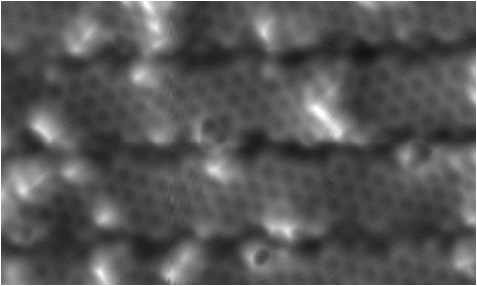

Supplement: Supplementary file 8 — Scanning probe microscopy images for Fig. 6. [file 41557_2022_1042_MOESM8_ESM.zip › Fig6g.png]

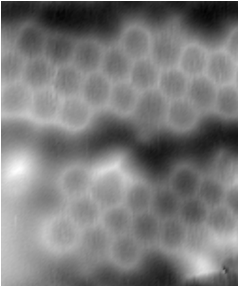

Supplement: Supplementary file 8 — Scanning probe microscopy images for Fig. 6. [file 41557_2022_1042_MOESM8_ESM.zip › Fig6h.png]

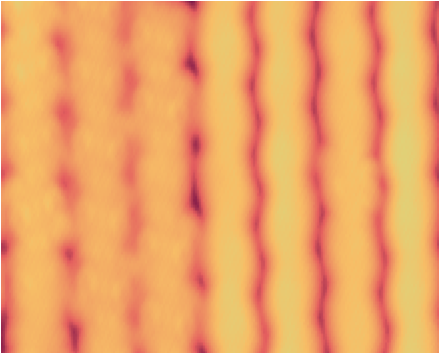

Supplement: Supplementary file 10 — Scanning probe microscopy images for Extended Data Fig. 2. [file 41557_2022_1042_MOESM10_ESM.zip › ExtDat2a.png]

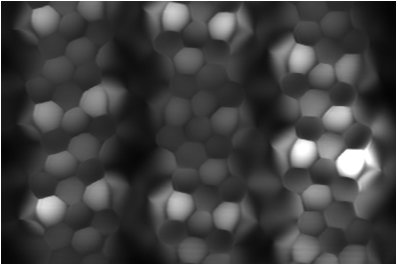

Supplement: Supplementary file 10 — Scanning probe microscopy images for Extended Data Fig. 2. [file 41557_2022_1042_MOESM10_ESM.zip › ExtDat2b.png]

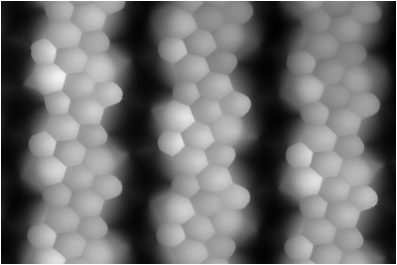

Supplement: Supplementary file 10 — Scanning probe microscopy images for Extended Data Fig. 2. [file 41557_2022_1042_MOESM10_ESM.zip › ExtDat2c.png]

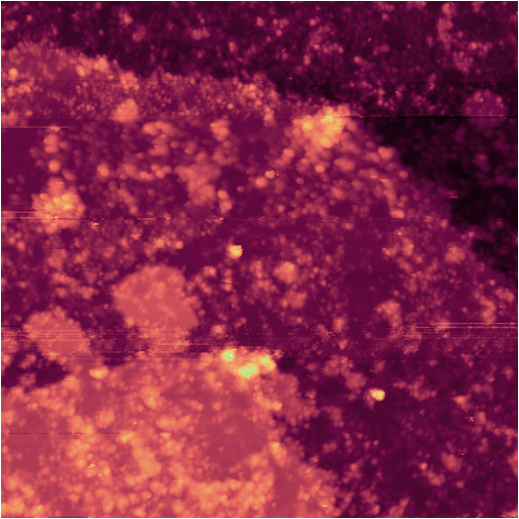

Supplement: Supplementary file 11 — Scanning probe microscopy images for Extended Data Fig. 3. [file 41557_2022_1042_MOESM11_ESM.zip › ExtDat3a.png]

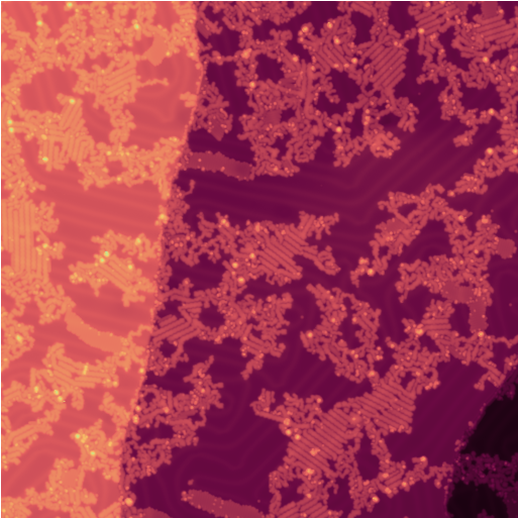

Supplement: Supplementary file 11 — Scanning probe microscopy images for Extended Data Fig. 3. [file 41557_2022_1042_MOESM11_ESM.zip › ExtDat3b.png]

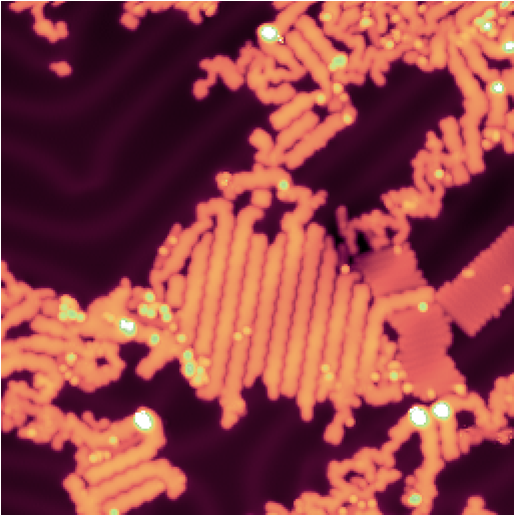

Supplement: Supplementary file 11 — Scanning probe microscopy images for Extended Data Fig. 3. [file 41557_2022_1042_MOESM11_ESM.zip › ExtDat3c.png]
